# Location‐scale models and cross validation to advance quantitative evidence synthesis

**DOI:** 10.1002/ecy.70303

**Published:** 2026-01-26

**Authors:** Shane A. Blowes

**Affiliations:** ^1^ German Centre for Integrative Biodiversity Research (iDiv) Halle‐Jena‐Leipzig Germany; ^2^ Department of Computer Science Martin Luther University Halle‐Wittenberg Halle (Saale) Germany

**Keywords:** biodiversity, habitat fragmentation, habitat loss, heterogeneity, heteroscedasticity, meta‐analysis, native‐exotic richness relationship, scale, transferability

## Abstract

Quantitative evidence synthesis is a prominent path towards generality in ecology. Generality is typically discussed in terms of central tendencies, such as an average effect across a compilation of studies, and the role of heterogeneity for assessing generality is less well developed. Heterogeneity examines the transferability of ecological effects across contexts, though between‐study variance is typically assumed as constant (i.e., homoscedastic). Here, I use two case studies to show how location‐scale models that relax the assumption of homoscedasticity and cross validation can combine to further the goals of evidence syntheses. First, I examine scale‐dependent heterogeneity for a meta‐analysis of plant native‐exotic species richness relationships, quantifying the relationships among unexplained effect size variation, spatial grain and extent. Second, I examine relationships among habitat fragment size, study‐level covariates and unexplained variation in patch‐scale species richness using a database of fragmentation studies. Heteroscedastic models quantify where effects can be transferred with more or less certainty and provide new descriptions of transferability for both case studies. Cross validation can be applied to a single or multiple models, adapted to either the goal of assessing intervention efficacy or generalization and, for the case studies examined here, showed that assuming homoscedasticity limits transferability.

## INTRODUCTION

Quantitative evidence synthesis aims for general insights into the direction, magnitude, and variability of ecological effects (Fox, 2019; Gurevitch et al., 2018; Spake et al., 2022). Two common forms of quantitative synthesis in ecology are meta‐analysis (i.e., analyses of effect sizes collated or calculated from existing studies) and analyses of primary data compilations (Mengersen et al., 2013; Spake et al., 2022). For both approaches, generalities typically come in the form of central tendencies, such as an average effect size across studies. While both approaches frequently quantify the heterogeneity of effect sizes across studies to examine how consistent ecological effects are (Gurevitch et al., 2018; Lau et al., 2013; Nakagawa, Yang, et al., 2023; Senior et al., 2016; Spake et al., 2022), the role of heterogeneity for assessing generality is less well developed.

Identifying sources of heterogeneity can be critical for understanding a given phenomenon (Gurevitch et al., 2018; Lau et al., 2013) and contributes to the goal of generality by determining how transferable effect sizes are across different contexts (Fox, 2019). However, effect size variance is typically assumed to be constant (i.e., homoscedastic; Nakagawa et al., 2025; Viechtbauer & López‐López, 2022, Williams et al., 2021), equating to an assumption that included studies sample a single statistical population (Williams et al., 2021). Moreover, assuming homoscedasticity limits questions to those focused on the magnitude of responses, whereas many questions would benefit from explicit (quantitative) examinations of variability (Cleasby & Nakagawa, 2011; Nakagawa et al., 2025). Location‐scale (mean–variance) models that can model heteroscedasticity have been described for both meta‐analytic models (Nakagawa et al., 2025; Viechtbauer & López‐López, 2022; Williams et al., 2021) and the multilevel or mixed‐effects models frequently used in syntheses of primary data (Lee & Nelder, 1996, 2006; Pinheiro & Bates, 2000; Zuur et al., 2009), but their use in quantitative evidence synthesis remains relatively rare (Nakagawa et al., 2025).

Specifying models for unexplained variation can provide direct assessments of the limits to transferability. For example, covariates associated with unexplained variation could help delineate contexts where quantitative evidence syntheses generalize with more or less uncertainty. If different groups, such as taxon groups or geographic regions, are associated with more or less unexplained variation, researchers could better communicate where effect sizes can be transferred with more or less confidence. Such quantitative assessments of unexplained variation can help identify promising directions for both empirical and theoretical research (Viechtbauer & López‐López, 2022).

To evaluate and/or compare homoscedastic and heteroscedastic models for evidence synthesis, some form of quantitative model testing is needed. Cross validation is a broadly applicable method used to evaluate model predictive performance (Hastie et al., 2009) and has the potential for direct assessments of transferability in an evidence synthesis context (Spake et al., 2022). Out‐of‐sample model predictions are a key component of transferability (Spake et al., 2022), and out‐of‐sample cross validation can be useful for a single model (e.g., does the model make more or less accurate predictions for different geographic regions or taxonomic groups?), as well as for comparing out‐of‐sample predictions between different models. Moreover, cross validation is highly flexible, and data splitting (Hastie et al., 2009) can be designed to compare the ability of models to make different types of predictions (e.g., Merkle et al., 2019; Yates et al., 2023). For example, an evidence synthesis with the goal of assessing the efficacy of an intervention (Gurevitch et al., 2018) might be best evaluated by testing within‐sample predictions, assuming included studies are a probability sample (Boyd et al., 2023) of the target group or population for the intervention, whereas, to meet the goal of generality (Fox, 2019; Gurevitch et al., 2018), models can be more usefully evaluated by their ability to predict data for a new study (or studies) outside of the data used to train the model.

To show how location‐scale models and cross validation can advance the goals of quantitative evidence synthesis in ecology, I present two case studies. Before the details of each case study, I briefly overview cross validation for evidence synthesis, introduce software capable of fitting location‐scale models, and describe the model fitting workflow. The first case study extends a meta‐analysis of spatial scale‐dependence in plant native‐exotic species richness relationships (Peng et al., 2019b) to quantify relationships among unexplained variation, grain size, and spatial extent. The second case study uses a primary data compilation of habitat fragment diversity studies (Chase et al., 2019). I focus on the relationship between fragment size and local (i.e., patch scale) species richness and examine whether residual variation is related to fragment size and other study‐level covariates. For both case studies, cross validation shows that the assumption of constant unexplained variation limits model predictive performance, especially when making predictions to new studies (i.e., out‐of‐sample predictions).

### Cross validation for model comparison in evidence synthesis

Cross validation uses data splitting techniques to test model predictive performance. Models are fit to a “training” data set and assessed on their ability to predict the “test” data set (Hastie et al., 2009). Cross validation requires a function to quantify predictive performance, and here I used pointwise *expected log predictive density* (*elpd*; see Yates et al., 2023 for further discussion of other common loss functions). Additionally, to minimize an inherent bias to support overfit models, I used the modified one‐standard‐error rule (Yates et al., 2021) that aims to select the least complex model that is comparable to the best scoring model.

To show how cross validation could be used to advance different goals of evidence synthesis (Gurevitch et al., 2018), I use cross validation to evaluate model performance for two different types of predictions: (1) within‐sample or conditional predictions that examine model performance when making predictions to new data within existing studies, and (2) out‐of‐sample or marginal predictions that assess predictive performance to new studies (Merkle et al., 2019; Yates et al., 2023).

To assess conditional (within‐sample) predictions, approximate or exact leave‐one‐out (loo) cross validation is considered the gold standard (Yates et al., 2023). However, approximate loo diagnostics showed many influential observations for the models in both case studies, which reduces the reliability of performance estimates (Vehtari et al., 2017), and because exact loo can be computationally expensive for large data compilations (due to the need to refit the model as many times as there are data points), I approximated loo using *k*‐fold cross validation. Importantly for cross validation, most quantitative evidence syntheses, including the case studies presented here, have dependencies in the data structure. For example, studies might contribute multiple effect sizes to a meta‐analysis, or the data might have spatial or other dependencies. Block cross validation (i.e., including structure in the sub‐setting process when portioning training and test data; Roberts et al., 2017; Yates et al., 2023) is typically recommended when the data themselves are structured. Accordingly, I use a stratified *k*‐fold approximation of loo for both case studies. Stratified *k*‐fold cross validation is appropriate for the hierarchical data typical of evidence syntheses because it balances data splitting among subgroups when creating the *k* blocks of data to which models are refit, ensuring that the relative group frequencies (here, the data coming from included studies) are maintained. Similar to loo, *k*‐fold cross validation is focused on making predictions to new data points within existing studies conditional on the model parameters, and I used *k* = 10 folds in both case studies to assess within‐sample predictive performance.

To assess out‐of‐sample predictive performance, I used leave‐one‐group‐out cross validation. Individual studies were removed one at a time, and models assessed on their ability to predict the data in the held‐out study.

### Software for fitting location‐scale models

Location‐scale statistical models allowing predictor variables for both the mean (location) and residual variation (scale) are well studied (Lee & Nelder, 1996, 2006; Pinheiro & Bates, 2000; Zuur et al., 2009) and have been described for meta‐analytic models (Nakagawa et al., 2025; Rodriguez et al., 2023; Viechtbauer & López‐López, 2022; Williams et al., 2021). Many R packages commonly used by ecologists allow for location‐scale models to be fit using either frequentist (e.g., *nlme*, Pinheiro & Bates, 2000; *glmmTMB*, Brooks et al., 2017, *metafor*, Viechtbauer, 2010) or Bayesian methods (e.g., *brms*, Bürkner, 2017); note that (as far as I am aware) frequentist packages are currently limited to fitting non‐varying (i.e., fixed) parameters for the scale component. Due to the availability of tools for diagnosing model pathologies (Betancourt, 2016; Monnahan et al., 2017), cross validation (Vehtari et al., 2017), and model calibration (Modrák et al., 2023), as well as the ability to fit varying (random) parameters for the scale component, I fit models using the Hamiltonian Monte Carlo (HMC) sampler Stan (Carpenter et al., 2017). Models were coded using the “brms” package (Bürkner, 2017). Code (and data) for all analyses is archived in Blowes (2025) at https://doi.org/10.5281/zenodo.17661443.

### Model fitting workflow

To robustly fit increasingly complex heteroscedastic models, I followed a workflow that used simulations to check the calibration of all models (Gelman et al., 2020; Modrák et al., 2023; Säilynoja et al., 2025; Talts et al., 2020). In particular, to examine whether inference with a particular model is feasible given the observed data, I focus on calibration in the parameter space of the empirical posterior (i.e., posterior simulation‐based calibration, Säilynoja et al., 2025). Briefly, the model is first fit to the empirical (i.e., observed) data. Then, the same model with priors informed by the fit to the empirical data is used to simulate many new data sets (with the same size, shape and structure as the empirical data), and the model is fit to each of the simulated data sets. Finally, plots examining model calibration (e.g., are known parameters of interest recovered with reasonable coverage?) are inspected. Appendix S1 presents the simulation‐based model calibrations for case study one; the calibration for models in case study two is in Appendix S2.

## CASE STUDY ONE: SCALE‐DEPENDENT UNCERTAINTY IN PLANT NATIVE‐EXOTIC RICHNESS RELATIONSHIPS

Communities with more species are often thought to be more resistant to invasion by exotic (or non‐native) species than communities with fewer species (Elton, 1958). However, the spatial scale‐dependence of biodiversity can complicate overly simple interpretations of this idea. Negative relationships between the numbers of native and non‐native species are thought more likely at small scales, with a switch to positive relationships expected at larger spatial scales (Levine, 2000). Such scale‐dependence has been linked to niche opportunities (Shea & Chesson, 2002). At relatively small scales, more species‐rich native communities leave fewer niche opportunities for exotics to invade. As spatial scale increases, so too does heterogeneity in resources, natural enemies, and the physical environment, which creates greater niche availability for exotic species to become established (Shea & Chesson, 2002).

Peng et al. (2019b) synthesized evidence for relationships between spatial scale (grain and extent) and the correlation of native and exotic species richness using multilevel meta‐regressions across 101 observational studies, encompassing 204 effect sizes. On average, Peng et al. (2019b) found native and exotic richness positively correlated, and that positive correlations between native and exotic species richness became stronger with increasing (log) grain size (i.e., the size of the sampling unit for the observations). All models fit by Peng et al. (2019b) assumed constant between‐study variance and adjusted for the nonindependence of multiple effect sizes coming from some studies with (nested) multilevel varying (random) intercepts. Here I relax the assumption that between‐study heterogeneity is constant, and examine whether unexplained variation in the effect size is related to either grain size or extent. I compare how all models perform for within and out‐of‐sample predictions using cross validation.

Peng et al. (2019b) transformed all correlations to Pearson product moment correlation coefficients (*r*) and calculated effect sizes using the Fisher *z*‐transformation: $z=\frac{1}{2}\log\left(\frac{1+r}{1-r}\right),$ with sampling (within‐case) variance estimated as $\mathrm{Var}\left(z\right)=\frac{1}{n-3}$ (denoted $s_{\mathrm{ij}}^{2}$ below); log refers to the natural logarithm (as it does hereafter), and *n* is the sample size (i.e., number of native‐exotic species pairs; effect size data sourced from Peng et al., 2019a). I start by reproducing the main result from Peng et al. (2019b) using a multilevel linear meta‐regression model for grain size that assumes the effect sizes, *z*_*ij*_, are normally distributed with known within‐case variance ($s_{\mathrm{ij}}^{2}$) and constant between‐study variance ($\tau^{2}$), which can be expressed as

(Model 1.1) $$\begin{array}{c} z_{\mathrm{ij}}\sim N\left(\mu_{\mathrm{ij}},s_{\mathrm{ij}}^{2}\right),\\ \mu_{\mathrm{ij}}=\beta_{0}+\beta_{0i}+\beta_{0\mathrm{ij}}+\beta_{1}X_{\mathrm{ij}},\\ \beta_{0i}\sim N\left(0,\tau^{2}\right),\;\beta_{0\mathrm{ij}}\sim N\left(0,\omega^{2}\right), \end{array}$$

where cases (*j*) are nested within studies (*i*) and have among‐case (within study) variance $\omega^{2}$; between‐study heterogeneity, $\beta_{0i}$, has constant variance $\tau^{2}$ and varies around the overall linear relationship for the location ($\mu_{\mathrm{ij}}$) with intercept $\beta_{0}$, slope $\beta_{1}$, and predictor $X_{\mathrm{ij}}$ (here the natural logarithm of grain size in study *i*, case *j*). Given the relationship between effect size and grain size (Peng et al., 2019b), I retain (log) grain size as a predictor in all subsequent models.

Peng et al. (2019b) observed that variation around the average (positive) relationship between grain size and effect sizes decreased with increasing grain size. That is, residual heteroscedasticity decreased as a function of grain size, though they did not explicitly model this relationship. Recall $s_{\mathrm{ij}}^{2}$ is the known (so‐called sampling) variance of the effect size estimate (and is not estimated from the data in any of the homoscedastic or heteroscedastic models presented here); the first heteroscedastic model introduces a new parameter $\left(\sigma_{\mathrm{ij}}^{2}\right)$ for the scale component of the model to be estimated from the data, and I model the (log) SD of this parameter (σ) as function of grain size:

(Model 1.2) $$\begin{array}{c} z_{\mathrm{ij}}\sim N\left(\mu_{\mathrm{ij}},s_{\mathrm{ij}}^{2}+\sigma_{\mathrm{ij}}^{2}\right),\\ \mu_{\mathrm{ij}}=\beta_{0}+\beta_{0i}+\beta_{1}X_{\mathrm{ij}},\\ \beta_{0i}\sim N\left(0,\tau^{2}\right),\log\left(\sigma_{\mathrm{ij}}\right)=\beta_{0}^{\sigma }+\beta_{1}^{\sigma }X_{\mathrm{ij}}, \end{array}$$

where *X*_*ij*_ is grain size on a log‐scale for the *j*th case in study *i*. This model did not converge when the case‐level random intercept (ω, see Model 1.1) was included, likely due to identification problems associated with multiple parameters (i.e., σ and ω) describing the same level of variation in the data.

To model extent, Peng et al. (2019b) used discrete bins with a range of one order of magnitude in square kilometers, that is, (0, 10), [10, 100), [10^2^, 10^3^), …, [10^6^, >10^6^), and multiple meta‐regression models were fit to examine for an effect of spatial extent on effect sizes. Peng et al. (2019b) did not report a strong influence of spatial extent on effect sizes, though they did describe an interaction between extent and grain. Here, I examine whether unexplained variation (i.e., after adjusting for the relationship between effect size and grain size) was related to extent using a model where residual variation was a function of spatial extent. I fit extent as a categorical predictor of the SD of residual variation ($\sigma_{\mathrm{ij}}$), that is, the same model for the scale as 1.2, but without the intercept ($\beta_{0}^{\omega }$), and extent categories coded as indicator variables for the predictor, *X*_*ij*_ (Model 1.3), instead of the continuous predictor fitted for grain in Model 1.2.

Finally, I fit a model that allowed between‐study variation in the scale component. Models with varying (or random) parameters specified for both the location and the scale components were first introduced as double hierarchical generalized linear models (Lee & Nelder, 1996, 2006), and they can be estimated with or without correlations among the varying parameters (see e.g., case study two below for an example with covarying parameters for the location and scale). The model was again fit with (log) grain size as a predictor ($X_{\mathrm{ij}}$) for the location (or mean effect size, $\mu_{\mathrm{ij}}$), and varying parameters for the scale were estimated independently of the location:

(Model 1.4) $$\begin{array}{c} z_{\mathrm{ij}}\sim N\left(\mu_{\mathrm{ij}},s_{\mathrm{ij}}^{2}+\sigma_{i}^{2}\right),\\ \mu_{\mathrm{ij}}=\beta_{0}+\beta_{0i}+\beta_{1}X_{\mathrm{ij}},\\ \beta_{0i}\sim N\left(0,\tau^{2}\right),\\ \log\left(\sigma_{i}\right)=\beta_{0}^{\sigma }+\beta_{0i}^{\sigma },\;\beta_{0i}^{\sigma }\sim N\left(0,\zeta^{2}\right), \end{array}$$

where $\beta_{0}^{\sigma }$ is the average residual SD (on a log‐scale), and $\beta_{0i}^{\sigma }$ is a normally distributed study‐level departure from the average intercept for the scale parameter with zero mean and ζ SD.

### Case study one: Results

All models reproduced the observed data well, and simulation‐based calibration showed all models had reasonable coverage for the parameters of interest (Appendix S1: Figures S1–S16).

**FIGURE 1:**
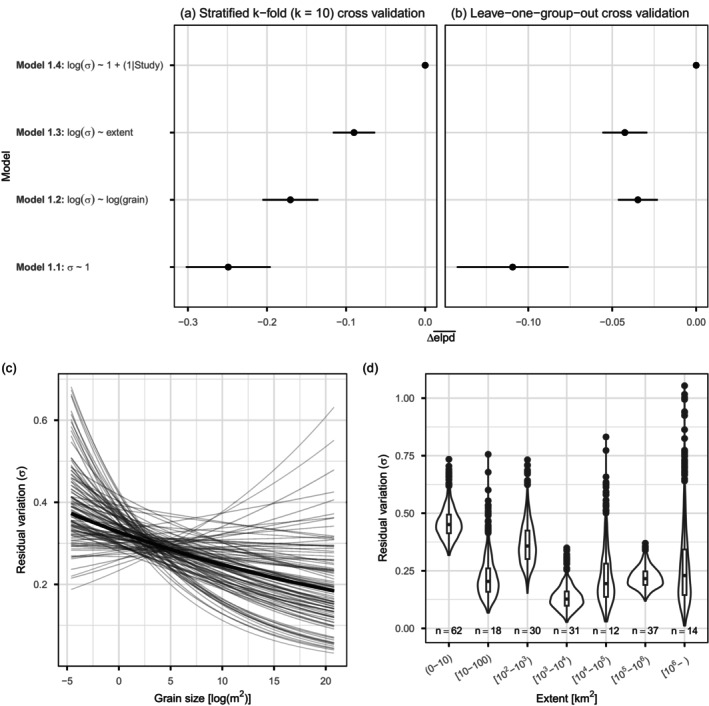
Heteroscedastic models outperform homoscedastic models for predicting plant native‐exotic species richness relationships. Model selection for predictions (a) within existing studies using stratified k‐fold cross validation and (b) for new studies (i.e., out‐of‐sample) using leave‐one‐group‐out cross validation. Scale‐dependent unexplained variation for (c) grain and (d) extent. Thick lines on (c) show median expectation for grain‐size‐dependent unexplained variation from Model 1.2 ($\log\left[\sigma_{\mathrm{ij}}\right]=\beta_{0}^{\sigma }$ + $\beta_{1}^{\sigma }X_{\mathrm{ij}}$); thin lines show 100 draws from the posterior distribution to visualize uncertainty. Violin plots (d) show the distribution of 1000 draws of the posterior distribution of unexplained variation (σ) for each of the extent categories; boxplots show the median (bar), 25% and 75% quantiles (box), whiskers show 1.5 times the interquartile range, points show observations beyond 1.5 times the interquartile range; *n* = number of effect sizes for each extent category (cases).

Model selection identified the same model (1.4) as best for making predictions within existing studies (Figure 1a) and to new studies (Figure 1b), though subsequent ordering among the other models depended on predictive task (Figure 1). All models produced similar estimates for the parameters they shared (i.e., β_0_, β_1_, τ; Appendix S1: Figure S17). The constant heterogeneity (i.e., homoscedastic) meta‐regression (Model 1.1) performed worst for making both within (Figure 1a), and out‐of‐sample predictions (Figure 1b). Model 1.2 quantified the average decrease in residual variation with increasing grain size (as was observed qualitatively by Peng et al., 2019b; $\beta_{1}^{\sigma }$= −0.03, 90% credible interval: −0.09 to 0.02) and showed uncertainty surrounding the relationship was greatest for the smallest and largest grains (Figure 1c). After adjusting for the positive relationship between average effect sizes (i.e., the location) and grain size, Model 1.3 showed unexplained variation was on average greatest at the smallest extents (i.e., the total geographic area covered by the samples; Figure 1d).

## CASE STUDY TWO: VARIATION IN PATCH‐SCALE EFFECTS OF ECOSYSTEM DECAY

Habitat loss is a major driver of biodiversity loss (Díaz et al., 2019). As habitat is removed from landscapes, biodiversity reductions in remaining habitat fragments can be thought of as arising from one of two processes (Chase et al., 2020). First, because fewer individuals and species can live in smaller habitat fragments, the species found in small habitat fragments might simply be a random (or passive) sample of those found in large habitat fragments. Alternatively, if smaller habitat fragments negatively impact demographic rates relative to larger habitat fragments, then ecosystem decay might result in greater diversity declines compared to those expected from a solely passive sampling process (Chase et al., 2020).

To test these competing hypotheses for (patch scale) diversity fragment size relationships, Chase et al. (2019) compiled assemblage data (counts of individuals of each species) in effort‐standardized (or standardizable) samples across habitat fragments of different sizes within landscapes. Initial analyses of these data showed that across 123 studies comprising 1509 habitat fragments, neither the number of individuals nor the species found in small fragments were simply a random sample of those in large habitat fragments (Chase et al., 2020). Instead, altered demographic rates in smaller habitat fragments, due, for example, to edge effects, reduced dispersal or demographic stochasticity (collectively referred to as ecosystem decay), resulted on average in fewer individuals, fewer species, and less even communities than expected from a passive sample of diversity in larger fragments (Chase et al., 2020). Here, I revisit this analysis to quantify heteroscedasticity and examine how modeling heteroscedasticity impacts within and out‐of‐sample model predictions. For simplicity, I focus on species richness and start by refitting the same multilevel model used by Chase et al. (2020) that assumed residual variation was constant across all of the studies. I then fit a series of models of increasing complexity that relax the assumption of homoscedasticity.

The homoscedastic model fit by Chase et al. (2020) to effort‐standardized species richness (*S*_*ij*_) in fragment *j* from study *i* took the form:

(Model 2.1) $$\begin{array}{c} S_{\mathrm{ij}}\sim \text{lognormal}\left(\mu_{\mathrm{ij}},\sigma^{2}\right)\\ \mu_{\mathrm{ij}}=\beta_{0}+\beta_{0i}+\left(\beta_{1}+\beta_{1i}\right)X_{\mathrm{ij}},\\ {\left[\beta_{0i},\beta_{1i}\right]}^{\prime }\sim \mathrm{MVN}\left(0,SRS\right),\\ S=\left[\begin{array}{cc} \sigma_{0i} & 0\\ 0 & \sigma_{1i} \end{array}\right],\;R=\left[\begin{array}{cc} 1 & \rho_{\sigma_{0i}\sigma_{1i}}\\ \rho_{\sigma_{0i}\sigma_{1i}} & 1 \end{array}\right], \end{array}$$

where *X*_*ij*_ is the fragment size on a (natural) log‐scale, which was centered by subtracting the overall mean from each observation before modeling; β_0i_ and β_1i_ are study‐level departures from the overall intercept ($\beta_{0}$) and slope ($\beta_{1}$), respectively, drawn from a multivariate normal (MVN) distribution with SDs, $\sigma_{0i}$ and $\sigma_{1i}$ that estimated correlations (ρ in the ***R*** matrix) between the varying intercepts and slopes for the location (i.e., mean richness, $\mu_{\mathrm{ij}}$).

Next, I consider heteroscedastic extensions of increasing complexity; all extensions take the form of double hierarchical generalized linear models (i.e., varying parameters for both the location and scale; Lee & Nelder, 1996, 2006). The first heteroscedastic model is motivated similarly to the varying study‐level intercepts and slopes for the location ($\mu_{\mathrm{ij}}$). Assuming average richness and its relationship with fragment size varies among studies gets us the benefit of adaptive regularization (i.e., shrinkage, McElreath, 2020) when estimating the location. And we can get the same types of benefits when estimating parameters for the scale (i.e., residual variation). Model 2.2 specified varying study‐level residuals estimated independently of other varying study‐level parameters for the location (mean):

(Model 2.2) $$\begin{array}{c} S_{\mathrm{ij}}\sim \text{lognormal}\left(\mu_{\mathrm{ij}},\sigma_{i}^{2}\right)\\ \mu_{\mathrm{ij}}=\beta_{0}+\beta_{0i}+\left(\beta_{1}+\beta_{1i}\right)X_{\mathrm{ij}},\\ {\left[\beta_{0i},\beta_{1i}\right]}^{\prime }\sim \mathrm{MVN}\left(0,SRS\right),\\ S=\left[\begin{array}{cc} \sigma_{0i} & 0\\ 0 & \sigma_{1i} \end{array}\right],\\ \;R=\left[\begin{array}{cc} 1 & \rho_{\sigma_{0i}\sigma_{1i}}\\ \rho_{\sigma_{0i}\sigma_{1i}} & 1 \end{array}\right],\\ \log\left(\sigma_{i}\right)=\beta_{0}^{\sigma }+\beta_{0i}^{\sigma },\;\beta_{0i}^{\sigma }\sim N\left(0,\sigma_{0i}^{\sigma }\right), \end{array}$$

where $\beta_{0}^{\sigma }$ is the overall average of residual variation (on a log‐scale) and $\beta_{0i}^{\sigma }$ are study‐level departures (for the scale or residual variation) drawn from a normal distribution with zero mean and SD $\sigma_{0i}^{\sigma }$.

Next, I extend this model to include (log) fragment size as a predictor of residual variation (i.e., scale). Correlations between varying (study level) intercepts and slopes of both the location and the scale are estimated, but varying parameters for the location (μ) are estimated independently of varying parameters for the scale (σ):

(Model 2.3) $$\begin{array}{c} S_{\mathrm{ij}}\sim \text{lognormal}\left(\mu_{\mathrm{ij}},\sigma_{i}^{2}\right)\\ \mu_{\mathrm{ij}}=\beta_{0}+\beta_{0i}+\left(\beta_{1}+\beta_{1i}\right)X_{\mathrm{ij}},\\ {\left[\beta_{0i},\beta_{1i}\right]}^{\prime }\sim \mathrm{MVN}\left(0,SRS\right),\\ S=\left[\begin{array}{cc} \sigma_{0i} & 0\\ 0 & \sigma_{1i} \end{array}\right],\\ R=\left[\begin{array}{cc} 1 & \rho_{\sigma_{0i}\sigma_{1i}}\\ \rho_{\sigma_{0i}\sigma_{1i}} & 1 \end{array}\right],\\ \log\left(\sigma_{i}\right)=\beta_{0}^{\sigma }+\beta_{0i}^{\sigma }+\left(\beta_{1}^{\sigma }+\beta_{1i}^{\sigma }\right)X_{i},\\ {\left[\beta_{0i}^{\sigma },\beta_{1i}^{\sigma }\right]}^{\prime }\sim \mathrm{MVN}\left(0,S^{\sigma }R^{\sigma }S^{\sigma }\right),\\ S^{\sigma }=\left[\begin{array}{cc} \sigma_{0i}^{\sigma } & 0\\ 0 & \sigma_{1i}^{\sigma } \end{array}\right],\;R^{\sigma }=\left[\begin{array}{cc} 1 & \rho_{\sigma_{0i}^{\sigma }\sigma_{1i}^{\sigma }}\\ \rho_{\sigma_{0i}^{\sigma }\sigma_{1i}^{\sigma }} & 1 \end{array}\right], \end{array}$$

where $\beta_{0}^{\sigma }$ is the overall average residual variation, and $\beta_{1}^{\sigma }$ is the overall average slope of residual variation with fragment size; $\beta_{0i}^{\sigma }$ and $\beta_{1i}^{\sigma }$ are the varying study‐level departures from the intercept and slope, respectively, and were drawn from a MVN distribution with zero mean and SD $\sigma_{0i}^{\sigma }$ and $\sigma_{1i}^{\sigma }$, with correlations estimated in matrix $R^{\sigma }$.

Finally, I estimate models with study‐level residual variation both with and without fragment size as a predictor that allows for correlations between varying (study level) parameters for the location (μ) and scale (σ). The model without fragment size is

(Model 2.4) $$\begin{array}{c} S_{\mathrm{ij}}\sim \text{lognormal}\left(\mu_{\mathrm{ij}},\sigma_{i}^{2}\right)\\ \mu_{\mathrm{ij}}=\beta_{0}+\beta_{0i}+\left(\beta_{1}+\beta_{1i}\right)X_{\mathrm{ij}},\\ \log\left(\sigma_{i}\right)=\beta_{0}^{\sigma }+\beta_{0i}^{\sigma },\\ {\left[\beta_{0i},\beta_{1i},\beta_{0i}^{\sigma }\right]}^{\prime }\sim \mathrm{MVN}\left(0,SRS\right),\\ S=\left[\begin{array}{ccc} \sigma_{0i} & 0 & 0\\ 0 & \sigma_{1i} & 0\\ 0 & 0 & \sigma_{0i}^{\sigma } \end{array}\right],\;R=\left[\begin{array}{ccc} 1 & \rho_{\sigma_{0i}\sigma_{1i}} & \rho_{\sigma_{0i}\sigma_{0i}^{\sigma }}\\ \rho_{\sigma_{0i}\sigma_{1i}} & 1 & \rho_{\sigma_{1i}\sigma_{0i}^{\sigma }}\\ \rho_{\sigma_{0i}\sigma_{0i}^{\sigma }} & \rho_{\sigma_{1i}\sigma_{0i}^{\sigma }} & 1 \end{array}\right]. \end{array}$$

The model with fragment size as a predictor of both the mean (location) and residuals (scale) that allows for correlations among the varying study‐level parameters is

(Model 2.5) $$\begin{array}{c} S_{\mathrm{ij}}\sim \text{lognormal}\left(\mu_{\mathrm{ij}},\sigma_{i}^{2}\right)\\ \mu_{\mathrm{ij}}=\beta_{0}+\beta_{0i}+\left(\beta_{1}+\beta_{1i}\right)X_{\mathrm{ij}},\\ \log\left(\sigma_{i}\right)=\beta_{0}^{\sigma }+\beta_{0i}^{\sigma }+\left(\beta_{1}^{\sigma }+\beta_{1i}^{\sigma }\right)X_{i},\\ {\left[\beta_{0i},\beta_{1i},\beta_{0i}^{\sigma },\beta_{1i}^{\sigma }\right]}^{\prime }\sim \mathrm{MVN}\left(0,SRS\right),\\ S=\left[\begin{array}{c} \sigma_{0i}\;0\;\begin{array}{cc} 0 & 0 \end{array}\\ \begin{array}{cc} 0 & \sigma_{1i} \end{array}\;\begin{array}{cc} 0 & 0 \end{array}\\ \begin{array}{cc} 0 & 0 \end{array}\;\begin{array}{cc} \sigma_{0i}^{\sigma } & 0 \end{array}\\ \begin{array}{cc} 0 & 0 \end{array}\;\begin{array}{cc} 0 & \sigma_{1i}^{\sigma } \end{array} \end{array}\right],\;R=\left[\begin{array}{c} \begin{array}{cc} 1 & \rho_{\sigma_{0i}\sigma_{1i}} \end{array}\;\begin{array}{cc} \rho_{\sigma_{0i}\sigma_{0i}^{\sigma }} & \rho_{\sigma_{0i}\sigma_{1i}^{\sigma }} \end{array}\\ \begin{array}{cc} \rho_{\sigma_{0i}\sigma_{1i}} & 1 \end{array}\;\begin{array}{cc} \rho_{\sigma_{1i}\sigma_{0i}^{\sigma }} & \rho_{\sigma_{1i}\sigma_{1i}^{\sigma }} \end{array}\\ \begin{array}{cc} \rho_{\sigma_{0i}\sigma_{0i}^{\sigma }} & \rho_{\sigma_{1i}\sigma_{0i}^{\sigma }} \end{array}\;\begin{array}{cc} 1 & \rho_{\sigma_{0i}^{\sigma }\sigma_{1i}^{\sigma }} \end{array}\\ \begin{array}{cc} \rho_{\sigma_{0i}\sigma_{1i}^{\sigma }} & \rho_{\sigma_{1i}\sigma_{1i}^{\sigma }} \end{array}\;\begin{array}{cc} \rho_{\sigma_{0i}^{\sigma }\sigma_{1i}^{\sigma }} & 1 \end{array} \end{array}\right]. \end{array}$$

### Case study two: Results

All models were able to reproduce the observed data well, and simulation‐based calibration showed that all models had reasonable coverage for parameters of interest (Appendix S2: Figures S1–S20).

**FIGURE 2:**
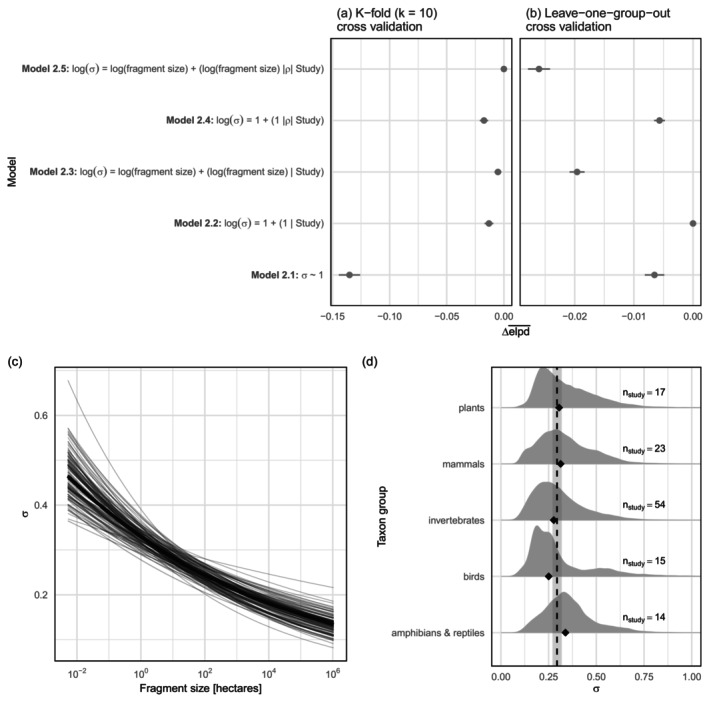
Patch‐scale species richness in habitat fragments is predicted best by models with heteroscedastic residual variation. Model selection for predictions (a) within existing studies using stratified k‐fold cross validation and (b) to new studies (i.e., out‐of‐sample) using leave‐one‐group‐out cross validation; (c) habitat fragment size‐dependent residual variation and (d) study‐level residual variation grouped into taxon groups. Bold line on (c) shows median predicted average relationship between fragment size and residual variation from Model 2.5, thin lines show 100 draws from the posterior distribution. Density plots (d) for taxon groups show study‐level variation (1000 draws from the posterior) of residual variation from Model 2.2 (i.e., $\beta_{0}^{\sigma }+\beta_{0i}^{\sigma }$ grouped into taxon groups); black triangle shows median σ for each taxon group, black‐dashed line and surrounding shading are the overall mean ($\beta_{0}^{\sigma }$) and 95% credible interval.

Modeling heteroscedasticity did not qualitatively impact the support for the ecosystem decay hypothesis for species richness (Appendix S2: Figure S21; Chase et al., 2020). However, heteroscedastic models outperformed the model with constant residual variation for making predictions of species richness for new fragments in existing studies (Figure 2a), and to entire new studies (Figure 2b), though different models were favored for within versus out‐of‐sample predictions. For making predictions to new data in existing studies, cross validation supported the most complex model (Model 2.5; Figure 2a). This model shows residual variation was a decreasing function of fragment size (Figure 2c) and estimated correlations between varying study‐level parameters for the location and scale (i.e., mean and residual variation; Appendix S2: Figure S22). For example, study‐level fragment size slopes for the mean effect and residual variation were negatively correlated (Appendix S2: Figure S22a), meaning that the strongest ecosystem decay effects were associated with the strongest decline in residual variation with increasing fragment size (Appendix S2: Figure S23).

For predictions to new studies leave‐one‐group‐out cross validation supported a simpler, multilevel model for residual variation (Figure 2b), where study‐level residuals varied around an overall mean (Model 2.2); each study gets its own (regularized) estimate of residual variation. As study encodes unique values for many other covariates (e.g., taxon group, matrix quality, time since fragmentation), this model can be used to further examine for systematic variation in the unexplained variation by plotting posterior samples of study‐level residual variation ($\sigma_{i}$) against study‐level covariates (see Chase et al., 2020 for a similar approach to examining predictors of average [location] ecosystem decay effect size estimates). For example, studies of amphibians and reptiles had more unexplained variation than average, while studies of birds had less unexplained variation than average (Figure 2d). The relatively small sample sizes (*n* = 14 and *n* = 15 studies, respectively) precludes strong inference, and I highlight this result to show: (1) explicit models of unexplained variation can help communicate where evidence for patch‐scale fragmentation effects are more or less uncertain and (2) as an example that the more nuanced description of the data provided by the heteroscedastic model can yield new insights for researchers to build on. For example, how do differences in connectivity between patches impact patch‐scale diversity variation?

## DISCUSSION

Quantitative evidence synthesis has become an increasingly prominent (Anderson et al., 2021) path towards generality for ecology (Fox, 2019; Gurevitch et al., 2018; Spake et al., 2022). Frequently, the heterogeneity of effects is quantified across different contexts (Gurevitch et al., 2018; Lau et al., 2013; Senior et al., 2016), providing insights into how transferable effects are (Fox, 2019). However, heterogeneity has typically been assumed to have constant variance, equivalent to an assumption of homoscedasticity (Nakagawa et al., 2025; Viechtbauer & López‐López, 2022; Williams et al., 2021). Here, I show how location‐scale models that relax the assumption of homoscedasticity can provide quantitative descriptions of where effect sizes can be transferred with more or less uncertainty, furthering the generalization goal of evidence synthesis. I also showed how cross validation can advance different goals of evidence synthesis.

Relaxing the assumption of homoscedasticity in meta‐analytic statistical models is relatively new (Nakagawa et al., 2025; Rodriguez et al., 2023; Viechtbauer & López‐López, 2022; Williams et al., 2021). This advent of location‐scale meta‐analytic models means that evidence syntheses of effects on variation (Cleasby & Nakagawa, 2011) are now possible using meta‐analytical models (Nakagawa et al., 2025, Viechtbauer & López‐López, 2022). For the meta‐analysis case study, all heteroscedastic models produced qualitatively similar estimates of parameters shared with the homoscedastic model (Appendix S1: Figure S3), though this will not always be the case (Williams et al., 2021). Here, heteroscedastic models quantified the observation of Peng et al. (2019b) that variation in native‐exotic plant species richness relationships decreases with increasing grain size and revealed considerable uncertainty for the smallest and largest grain sizes (Figure 1c). Moreover, heteroscedastic models showed that the smallest extents had the most unexplained variation on average, suggesting that there is considerable context‐dependency in plant native‐exotic richness relationships at the smallest spatial scales (i.e., small grains and small extents).

Location‐scale models have a longer history for statistical models fit to primary data (Lee & Nelder, 1996, 2006). Heteroscedastic models for the relationship between patch‐scale species richness and fragment size revealed previously undescribed patterns of variation: Smaller habitat fragments exhibit greater richness variation than larger habitat fragments. Some of this relationship might be due to the typically few large fragments sampled within landscapes (i.e., the data typically have less scope for residual variation among large fragments). Yet ecological variation is also possible. For example, small habitat fragments could experience greater variation in processes associated with ecosystem decay (e.g., demographic stochasticity, edge effects) than larger fragments. Indeed, the most complex heteroscedastic model showed that the strongest ecosystem decay effects on average patch‐scale richness were accompanied by the fastest decline in (residual) variation with increasing fragment size (Appendix S2: Figures S22a and S23). This suggests that processes associated with ecosystem decay could be amplifying patch‐scale richness variation in small habitat fragments.

To date, model selection in evidence synthesis has typically used either variance explained (*R* ^2^, Nakagawa, Yang, et al., 2023) or information criterion methods (Cinar et al., 2021). Here, I introduced cross validation as a flexible alternative. Cross validation can be used to evaluate a single model, or to compare multiple models. Moreover, cross validation can be used to assess different types of predictions, such as the within versus out‐of‐sample predictive performance compared in the case studies here. This means cross validation can be tailored for the different goals an evidence synthesis might have (Gurevitch et al., 2018), and the case studies presented here showed that the ranking of models can depend on the predictive task. Within‐sample predictions are likely most suited to evidence syntheses where the goal is predicting the success (or efficacy) of an intervention (Gurevitch et al., 2018), assuming that the compiled data are a representative sample of the population targeted for intervention (Boyd et al., 2023). However, it is important to note that neither case study presented here had the goal of assessing intervention efficacy.

Evidence syntheses in ecology are more typically seeking broad generalizations (Gurevitch et al., 2018). For this goal, cross validation tests of out‐of‐sample predictions can provide direct evidence for how transferable model predictions are to different contexts (Spake et al., 2022). Here, I used leave‐one‐group‐out cross validation to assess out‐of‐sample predictions with the simplest (and most general) grouping structure typical of an evidence synthesis, that is, predictions to a single new study. However, studies could be further grouped, for example, by taxonomic group, (bio)geographically, or phylogenetically, and combined with a different loss function for easier interpretation (e.g., mean squared or absolute error; Yates et al., 2023) to provide a more constrained test of transferability for a single model. When comparing models, both case studies found that a model with study‐level variation for the scale (residual) component was best for out‐of‐sample predictions, suggesting this so‐called double hierarchical model (Lee & Nelder, 1996, 2006) might be a good starting point for most evidence syntheses (Nakagawa et al., 2025), particularly where the goal is generalization.

Heteroscedastic models add complexity to analyses. Here, I used simulation‐based calibration (Gelman et al., 2020; Modrák et al., 2023; Talts et al., 2020) to validate all of the models fit in both case studies, with a focus on parameter space conditioned on the observed data (i.e., posterior simulation‐based calibration; Säilynoja et al., 2025). Posterior simulation‐based calibration is particularly suited to ecological models fit to empirical data, as it improves confidence in model‐based inferences by validating and visualizing uncertainty for known parameter values in the posterior region of the observed data (Säilynoja et al., 2025).

Location‐scale statistical models and cross validation promise to strengthen quantitative evidence synthesis in ecology. Location‐scale models will help communicate where effects can be transferred with more or less uncertainty and broaden the scope of questions to include variability of effects. Cross validation can be tailored to meet the common goals of evidence synthesis. For the case studies here, heteroscedastic models were favored for making out‐of‐sample predictions (i.e., to a new study) and showed location‐scale models and cross validation can combine to provide new insights for ecological evidence syntheses seeking generalities.

## CONFLICT OF INTEREST STATEMENT

The author declares no conflicts of interest.

## Supporting information


Appendix S1.

Appendix S2.

## Data Availability

Data used in this work are available in Dryad (Peng et al., 2019a; https://doi.org/10.5061/dryad.59kv753) and in *Ecology* (Chase et al., 2019; https://doi.org/10.1002/ecy.2861). Code (Blowes, 2025) is available in Zenodo at https://doi.org/10.5281/zenodo.17661443.
