# Supplementary material for: Location‐scale models and cross validation to advance quantitative evidence synthesis
Source: Ecology. 2026 Jan 26;107(1):e70303. doi: 10.1002/ecy.70303 (PMC12834672; doi:10.1002/ecy.70303)
Supplement: Supplementary file 1 — Appendix S1. [file ECY-107-e70303-s002.pdf]

Appendix S1

Author: Shane Blowes

Title: Location-scale models and cross validation to advance quantitative evidence synthesis

Journal: Ecology

Case-study one: Simulation-based calibration and supplemental figures

I used Simulation-Based Calibration (Talts et al. 2020, Modrák et al. 2023, Säilynoja et al. 2025) to check whether models could recover known parameter values with reasonable coverage (i.e., the probability that a constructed [e.g., credible] interval contains the true value), accuracy and precision. I focus calibration on the region of the parameter space around the empirical posterior (i.e., posterior simulation-based calibration; Säilynoja et al. 2025). Briefly, this involves: (1) fitting a model to the empirical data; (2) using the same model with priors informed by the fit to the empirical data to simulate many new (fake) datasets with the same size, shape and structure as the empirical data (i.e., the same number of observations, and the same number of groups for each level in the hierarchical structure); (3) refitting the model to each simulated data set; and, (4) calculating SBC diagnostics and plotting results of the model fits to simulated data to check for reasonable coverage (Talts et al. 2020, Modrák et al. 2023, Säilynoja et al. 2025).

To examine model calibration, I focus on three plots from the SBC diagnostics: (1) a histogram of the posterior ranks of the prior draws, which (if the algorithm and model are working correctly) should be approximately normally distributed; (2) the empirical coverage of parameters of interest (coverage is the proportion of known variable values that fall within the interval: a well calibrated model would have coverage exactly matching the interval width, e.g., 50% credible interval contains the known value 50% of the time); and, (3) a plot of estimated

parameter values as a function of known (simulated) parameter values for parameters of interest, which shows how accurately and precisely the model estimates focal parameters.

Model 1.1

I begin case study one by reproducing the main result from Peng et al. (2019) using a multilevel linear meta-regression model for grain size that assumes the effect sizes, z_{ij}, are normally distributed with known within-case variance (s_{ij}^2) and constant between-study variance (τ^2), which can be expressed as:

$$z_{ij} \sim N(\mu_{ij}, s_{ij}^2),$$

$$\mu_{ij} = \beta_0 + \beta_{0i} + \beta_{0ij} + \beta_1 X_{ij},$$

$$\beta_{0i} \sim N(0, \tau^2),$$

$$\beta_{0ij} \sim N(0, \omega^2),$$

where cases (*j*) are nested within studies (*i*), and have among-case (within study) variance, ω^2; study-level heterogeneity, β_{0i}, has variance τ^2, and varies around the overall linear relationship with intercept, β_0, slope β_1, and predictor X_i (here the natural logarithm of grain size in study *i*). The model was fit to empirical data with weakly regularizing priors:

$$\beta_0 \sim N(0.3, 1),$$

$$\beta_1 \sim N(0, 1),$$

$$[\tau, \omega] \sim N(0, 1), \qquad \text{(Model 1.1)}.$$

This model had good convergence (all Rhat < 1.01), and showed a reasonable fit to the empirical data (Appendix S2: Fig. S1).

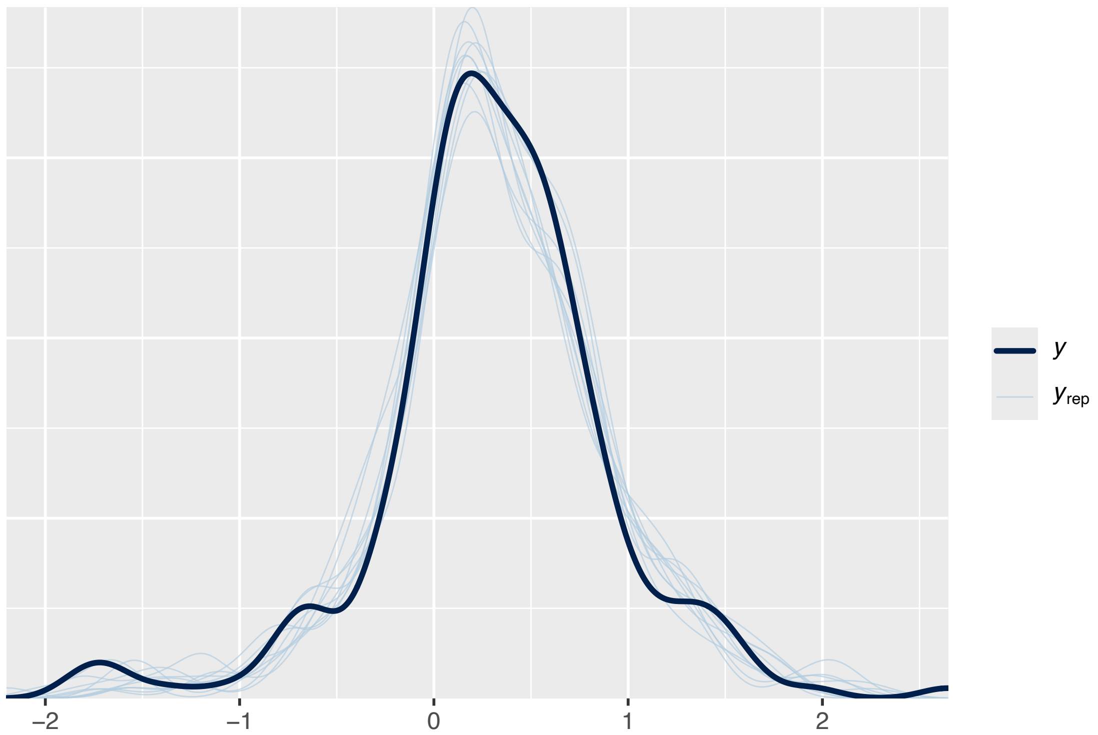

Figure S1: Posterior predictive check for model 1.1 fit to the empirical data (*z*-scores).

The parameter estimates from the fit of model 1.1 to empirical data (β_0: 0.13 [95% credible interval: 0.01 – 0.24]; β_1: 0.04 [95% credible interval: 0.02 – 0.05]; τ: 0.34 [95% credible interval: 0.25 – 0.43]; ω: 0.3 [95% credible interval: 0.24 – 0.35]) were used to inform the following priors:

$$\beta_0 \sim N(0.13, 0.06),$$

$$\beta_1 \sim N(0.04, 0.02),$$

$$\tau \sim N(0, 0.25),$$

$$\omega \sim N(0, 0.25),$$

which were combined with model 1.1 to simulate (fake) data sets. To ensure as much realism as possible in the simulated data, each simulated data set retained the characteristics of the empirical data: 204 observations distributed across 101 studies (with the same balance, i.e., effect sizes per study, and the known standard errors $[s_{ij}]$ from the empirical data). I refit model

1.1 to each simulated data set, and identified models fit to fake data with poor diagnostics (e.g., divergent transitions and Rhats > 1.05); I report the number of simulated data sets used to calculate simulation diagnostics in figure captions.

Simulation-based calibration for model 1.1 showed that the rank statistics were approximately uniformly distributed (Appendix S2: Fig. S2), and that the coverage of parameters was reasonable (Appendix S2: Fig. S3); known parameters were approximately recovered (Appendix S2: Fig. S4).

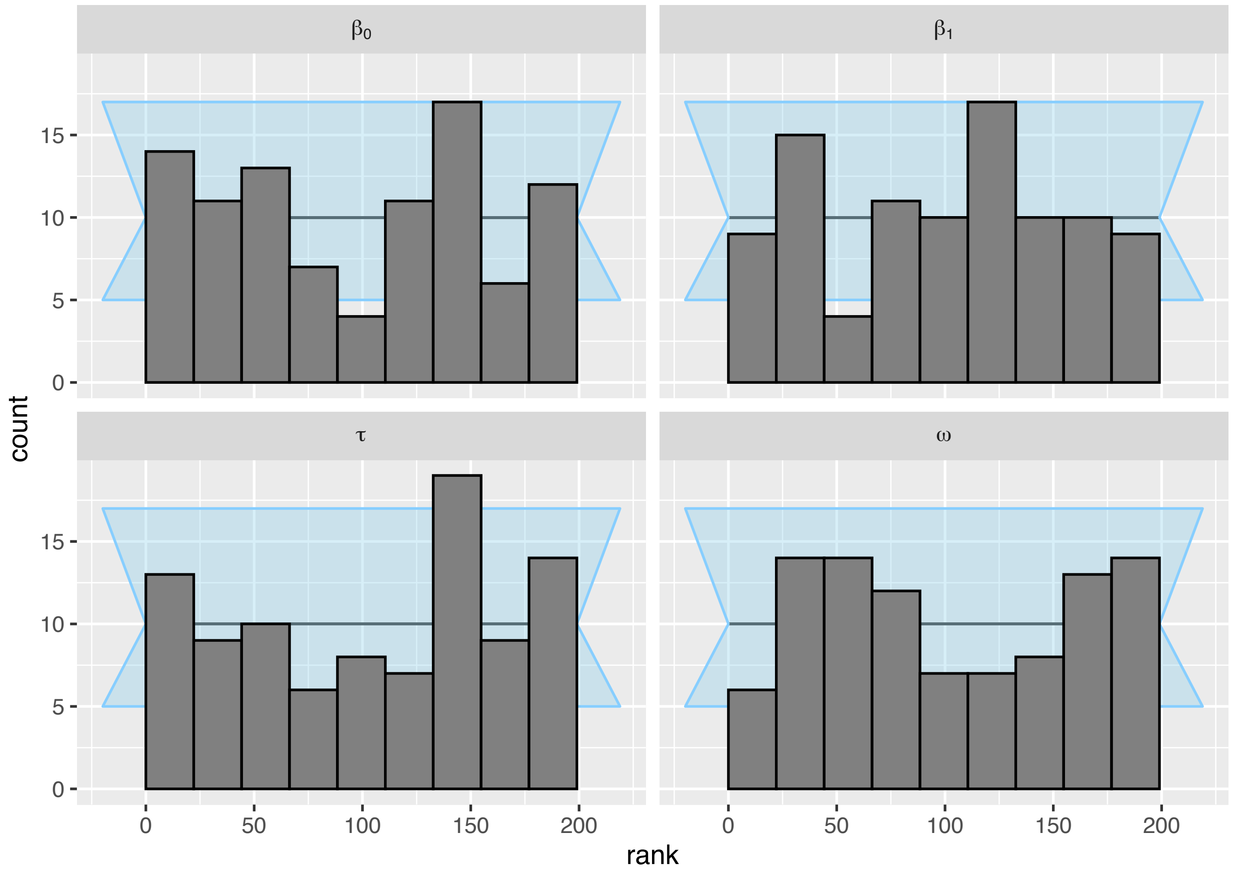

Figure S2: The posterior ranks of the prior draws were approximately normally distributed for the parameters of interest in model 1.1. Results are shown for n = 95 simulated data sets. Background (light blue shading) shows an approximate 95% interval for expected deviations.

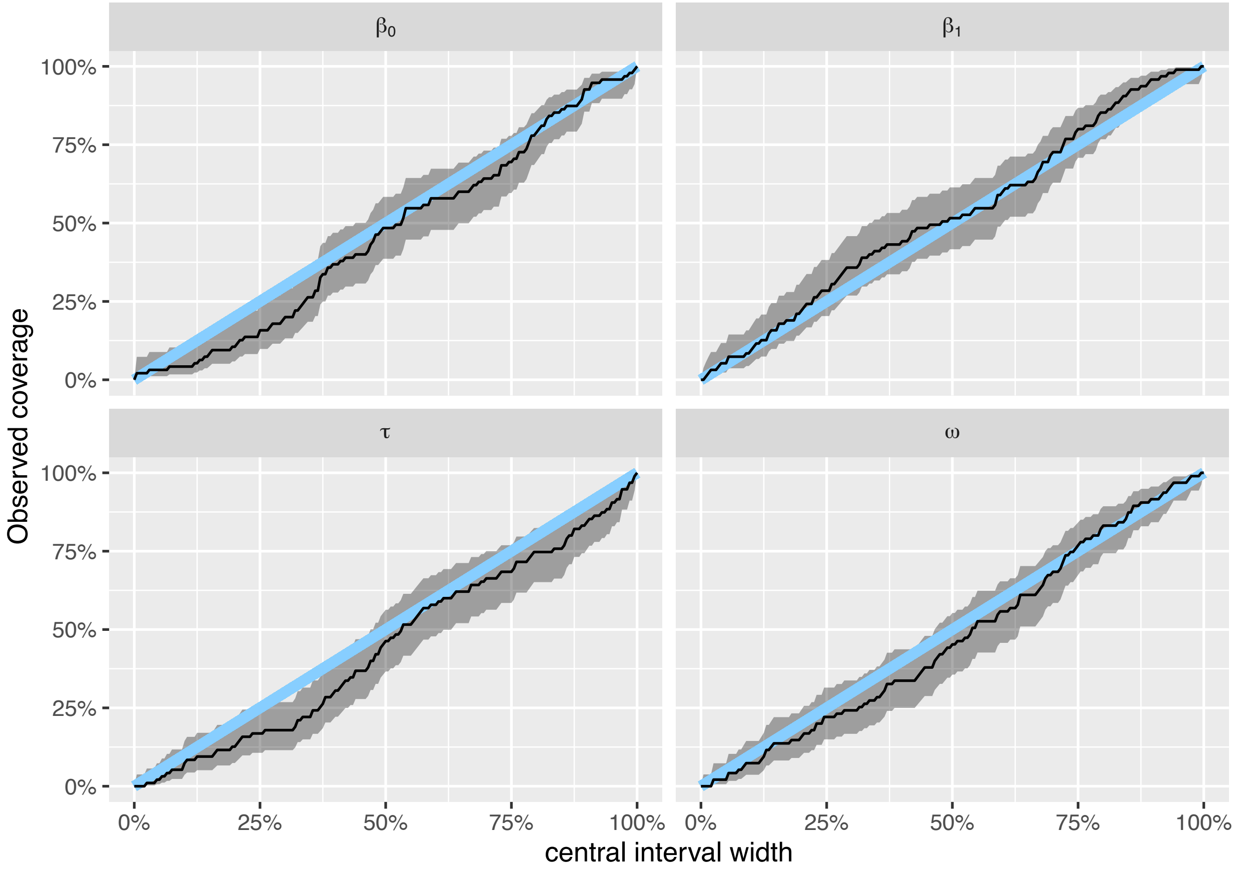

Figure S3: Model 1.1 had reasonable coverage for the parameters of interest. Results are shown for n = 95 simulated data sets. Blue line is 1:1 line, and shading shows 95% uncertainty interval for the coverage.

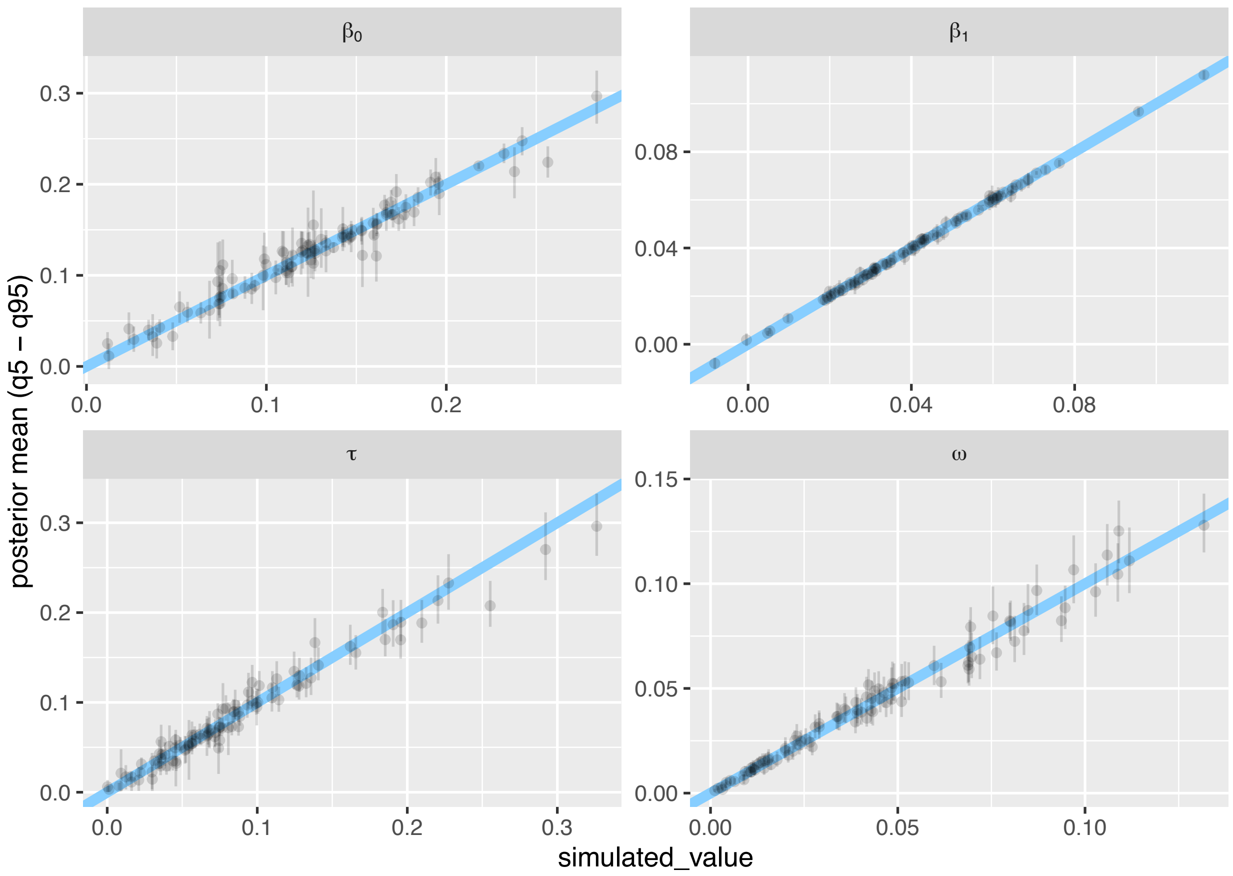

Figure S4: Model 1.1 was largely able to recover known parameter values. Results are shown for n = 95 simulated data sets; "simulated_value" (x-axis) is the known value of the parameter for a given simulation. Each point shows a parameter estimate, whiskers show 95% credible interval; diagonal line is the 1:1 line.

Model 1.2

The second model in case study one extended model 1.1 to include an additional parameter (σ_{ij}) for residual variation as a function of grain size:

$$z_{ij} \sim N(\mu_{ij}, s_{ij}^2 + \sigma_{ij}^2),$$

$$\mu_{ij} = \beta_0 + \beta_{0i} + \beta_1 X_i,$$

$$\beta_{0i} \sim N(0, \tau^2),$$

$$log(\sigma_{ij}) = \beta_0^\sigma + \beta_1^\sigma X_{ij},$$

where X_{ij} is grain size on a log-scale for the *j*th case in study *i*. I fit this model to the empirical data with weakly regularizing priors:

$$\beta_0 \sim N(0.3, 1),$$

$$\beta_0^\sigma, \beta_1^\sigma, \beta_1 \sim N(0, 1),$$

$$\tau \sim N(0, 1), \qquad \text{(Model 1.2).}$$

Model 1.2 had good convergence (all Rhats < 1.01), and showed a good fit to the empirical data (Appendix S2: Fig. S5).

The parameter estimates from the fit of model 1.2 to empirical data (β_0: 0.13 [95% credible interval: 0.01 – 0.24]; β_1: 0.04 [95% credible interval: 0.02 – 0.05]; τ: 0.33 [95% credible interval: 0.24 – 0.42]; β_0^σ: -1.12 [95% credible interval: -1.4 – -0.86]; β_1^σ: -0.03 [95% credible interval: -0.1 – 0.03]) were used to inform the following priors:

$$\beta_0 \sim N(0.13, 0.06),$$

$$\beta_1 \sim N(0.04, 0.02),$$

$$\tau \sim N(0, 0.2),$$

$$\beta_0^\sigma \sim N(-1.1, 0.2),$$

$$\beta_1^\sigma \sim N(-0.03, 0.03),$$

which were combined with model 1.2 to simulate (fake) data sets.

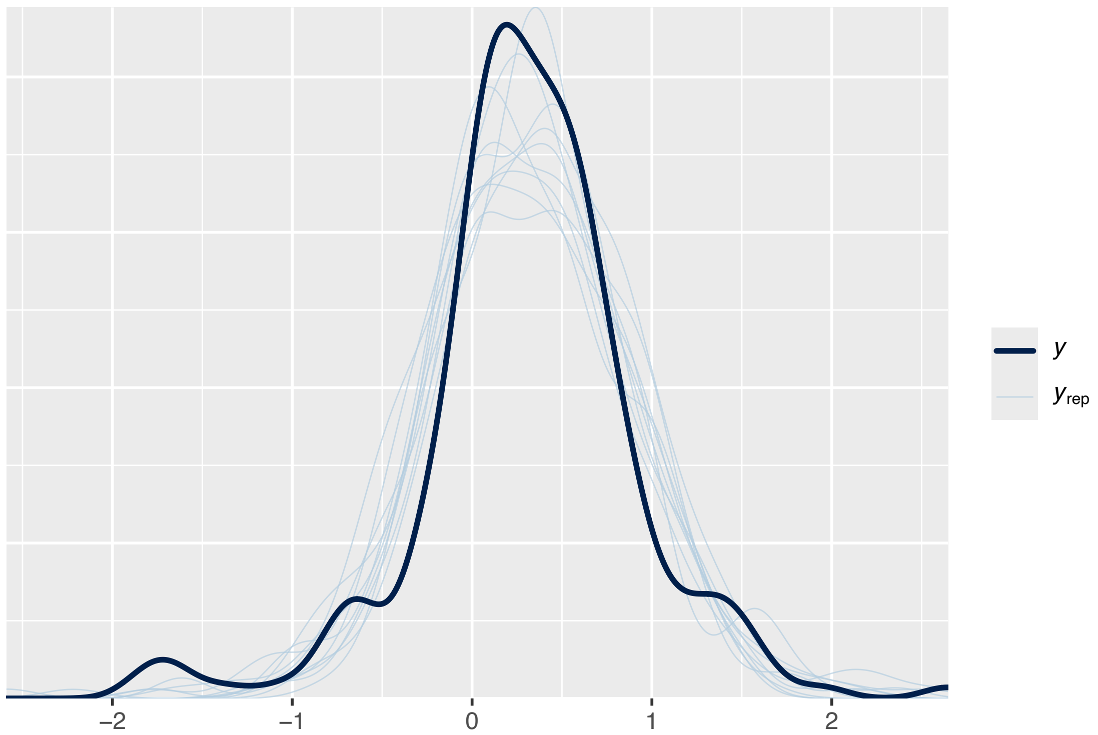

Figure S5: Posterior predictions from model 1.2 were largely consistent with the observed data.

Simulation-based calibration for model 1.2 showed that the rank statistics were approximately uniformly distributed (Appendix S2: Fig. S6), and that the coverage of parameters was reasonable (Appendix S2: Fig. S7); known parameters were approximately recovered, though with a fair amount of uncertainty for the intercept (β_0), the varying intercept for studies (τ), and the slope of relationship between unexplained variation and grain size (β_1^{σ}; Appendix S2: Fig. S8).

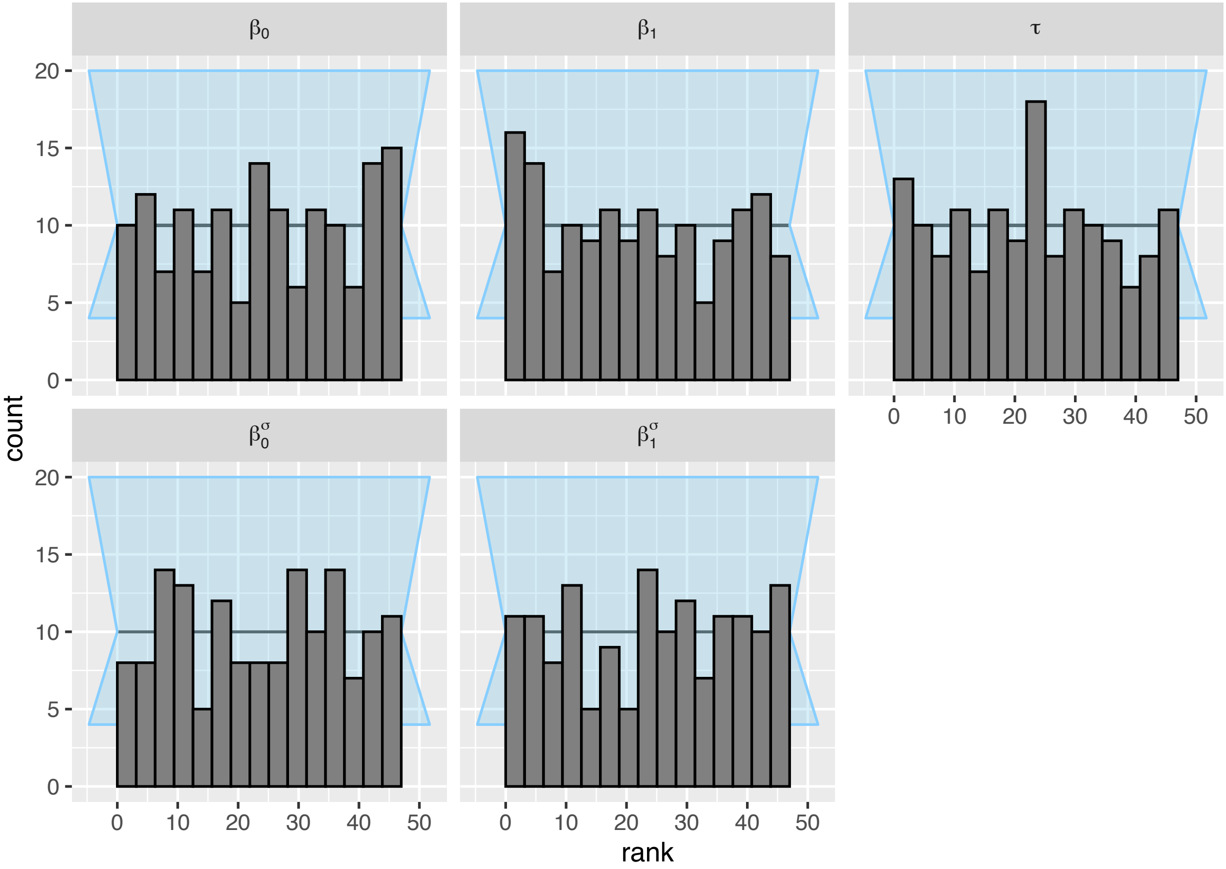

Figure S6: The posterior ranks of the prior draws were approximately normally distributed for the parameters of interest in model 1.2. Results are shown for n = 150 simulated data sets. Background (light blue shading) shows an approximate 95% interval for expected deviations.

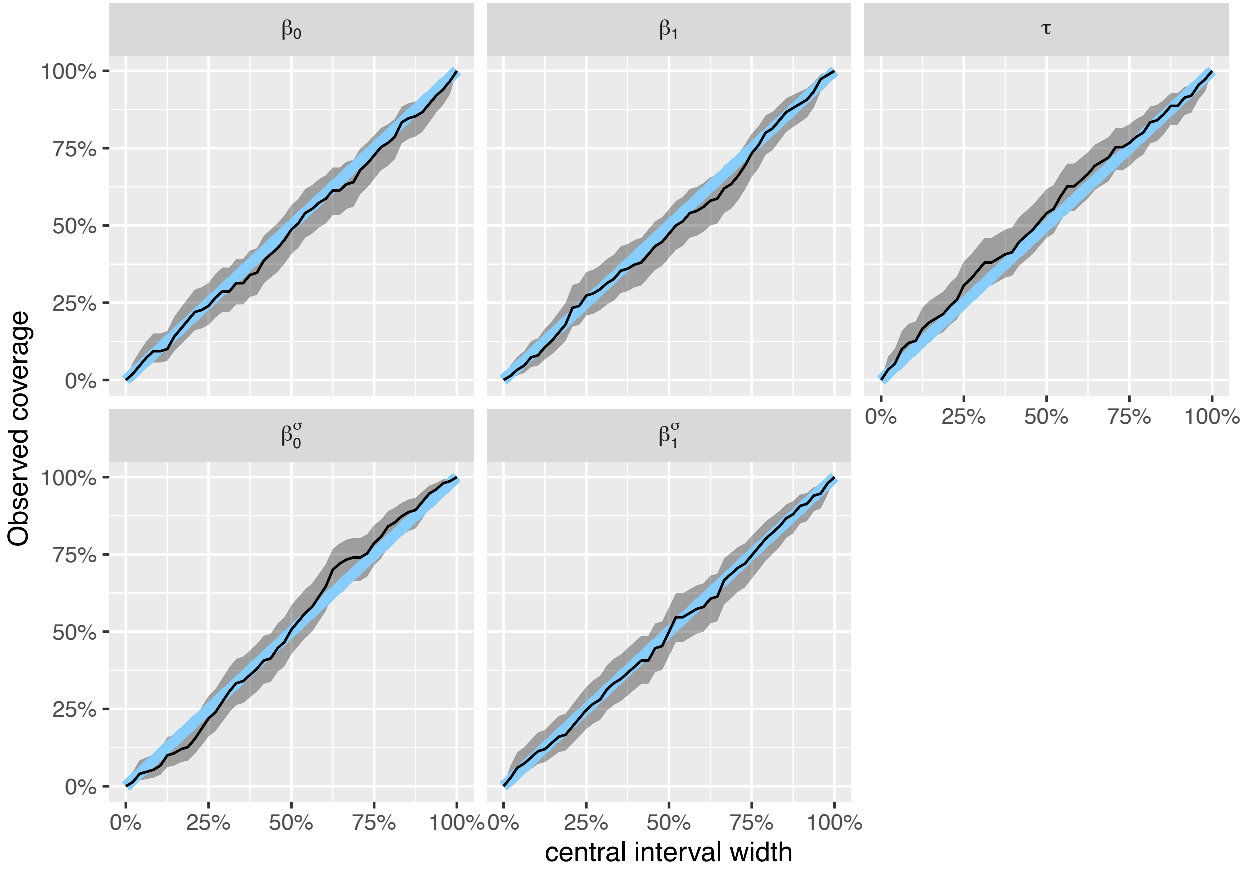

Figure S7: Model 1.2 had reasonable coverage for the parameters of interest. Results are shown for n = 150 simulated data sets. Blue line is 1:1 line, and shading shows 95% uncertainty interval for the coverage.

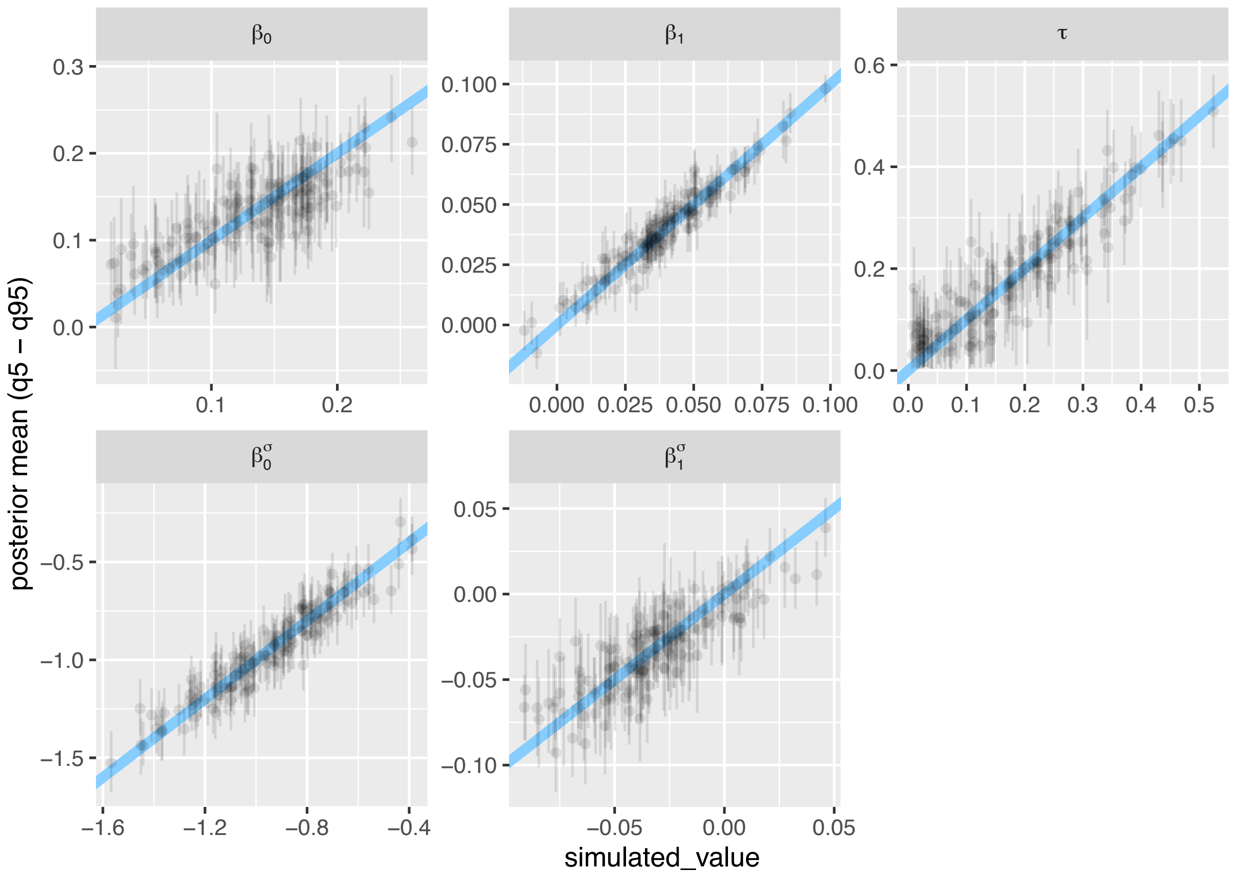

Figure S8: Model 1.2 was able to recover known parameter values, though with some uncertainty (especially for β_0, τ, and β_1^σ). Results are shown for n = 150 simulated data sets; "simulated_value" (x-axis) is the known value of the parameter for a given simulation. Each point shows a parameter estimate, whiskers show 95% credible interval; diagonal line is the 1:1 line.

Model 1.3

The third model replaced grain size as a predictor of unexplained variation with spatial extent, which was estimated with indicator variables for each category of spatial extent [i.e., no intercept, see main text]). I fit this model to the empirical data with the same weakly regularizing priors as model 1.2 (with the exception of β_0^σ, which was dropped from the model).

Model 1.3 had good convergence (all Rhats < 1.01), and showed a reasonable fit to the empirical data (Appendix S2: Fig. S9).

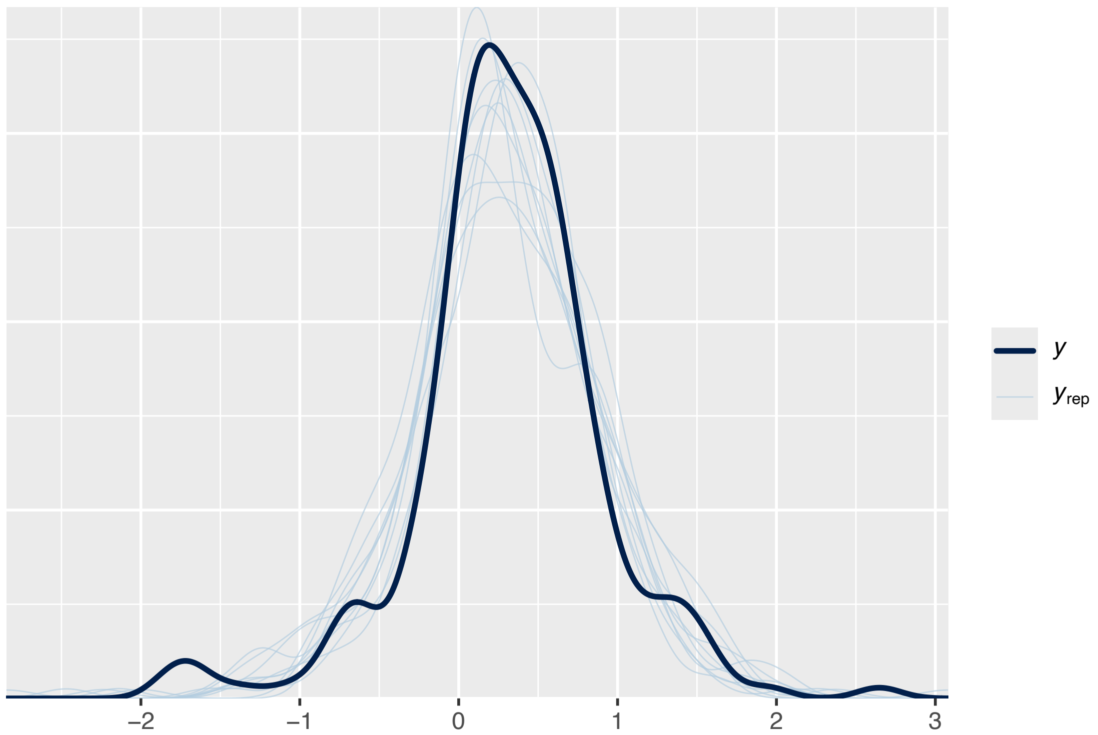

Figure S9: Posterior predictions for model 1.3 showed reasonable fidelity to the empirical data.

The parameter estimates from the fit of model 1.3 to empirical data (β_0: 0.17 [95% credible interval: 0.06 – 0.28]; β_1: 0.03 [95% credible interval: 0.02 – 0.04]; τ: 0.34 [95% credible interval: 0.25 – 0.43]; β^{σ}_{0-10}: -0.79 [95% credible interval: -1.07 - -0.52]; β^{σ}_{10-100}: -1.58 [95% credible interval: -2.3 - -0.83]; $\beta^{\sigma}_{10^{\wedge}2-10^{\wedge}3}$: -1.03 [95% credible interval: -1.5 - -0.57]; $\beta^{\sigma}_{10^{\wedge}3-10^{\wedge}4}$: -2.09 [95% credible interval: -2.95- -1.39]; $\beta^{\sigma}_{10^{\wedge}4-10^{\wedge}5}$: -1.66 [95% credible interval: -2.93 - -0.69]; $\beta^{\sigma}_{10^{\wedge}5-10^{\wedge}6}$: -1.53 [95% credible interval: -1.91 - -1.13]; $\beta^{\sigma}_{10^{\wedge}6-}$: -1.51 [95% credible interval: -2.87- -0.38]) were used to inform the following priors:

$$\beta_0 \sim N(0.13, 0.05),$$

$$\beta_1 \sim N(0.04, 0.02),$$

$$\tau \sim N(0, 0.2),$$

$$\beta^{\sigma}_1 \sim N(-1,1),$$

which were combined with model 1.3 to simulate (fake) data sets.

Simulation-based calibration for model 1.3 showed that the rank statistics were approximately uniformly distributed (Appendix S2: Fig. S10), and that the coverage of parameters was reasonable (Appendix S2: Fig. S11); known parameters were approximately recovered, though with a fair amount of uncertainty for the intercept (β_0), and the varying intercept for studies (τ, Appendix S2: Fig. S12).

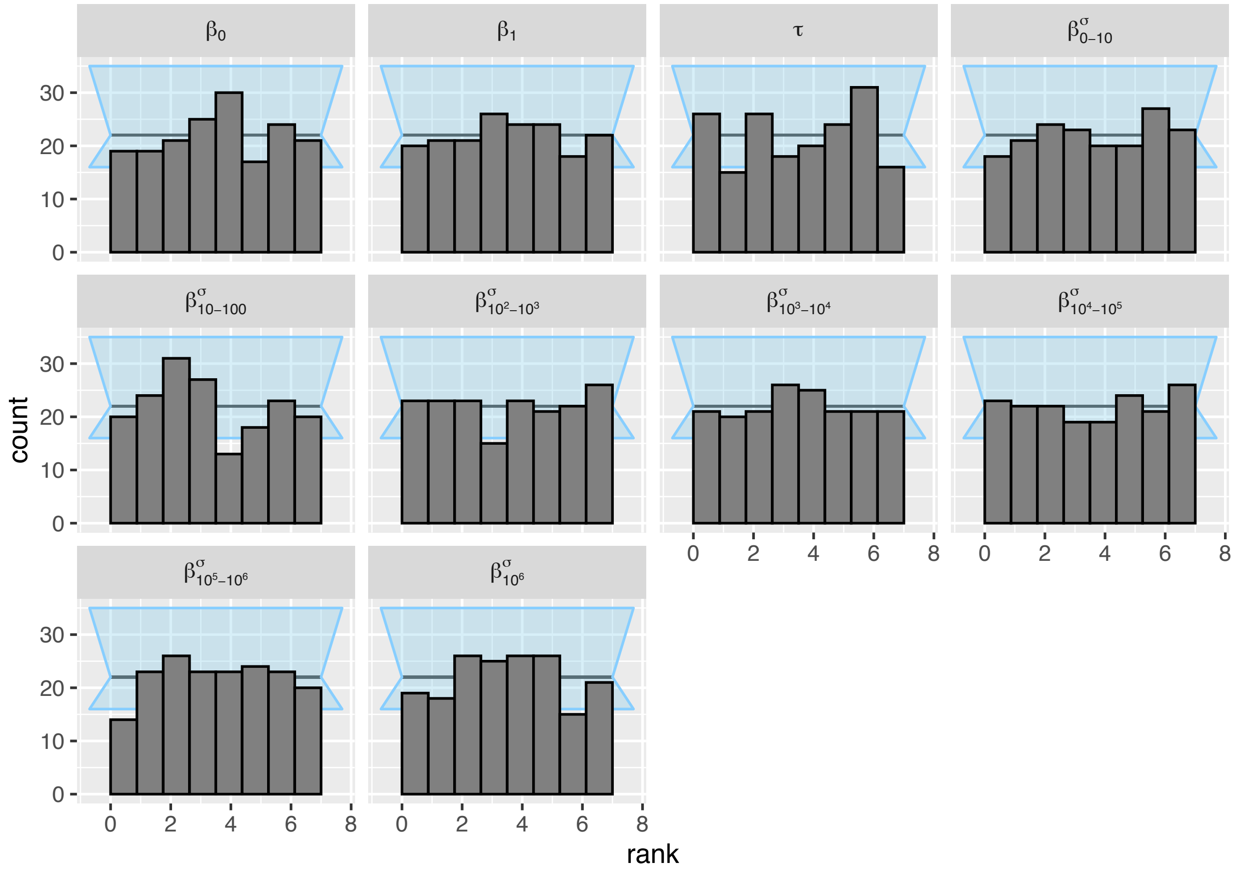

Figure S10: The posterior ranks of the prior draws were approximately normally distributed for the parameters of interest in model 1.3. Results are shown for n = 176 simulated data sets. Background (light blue shading) shows an approximate 95% interval for expected deviations.

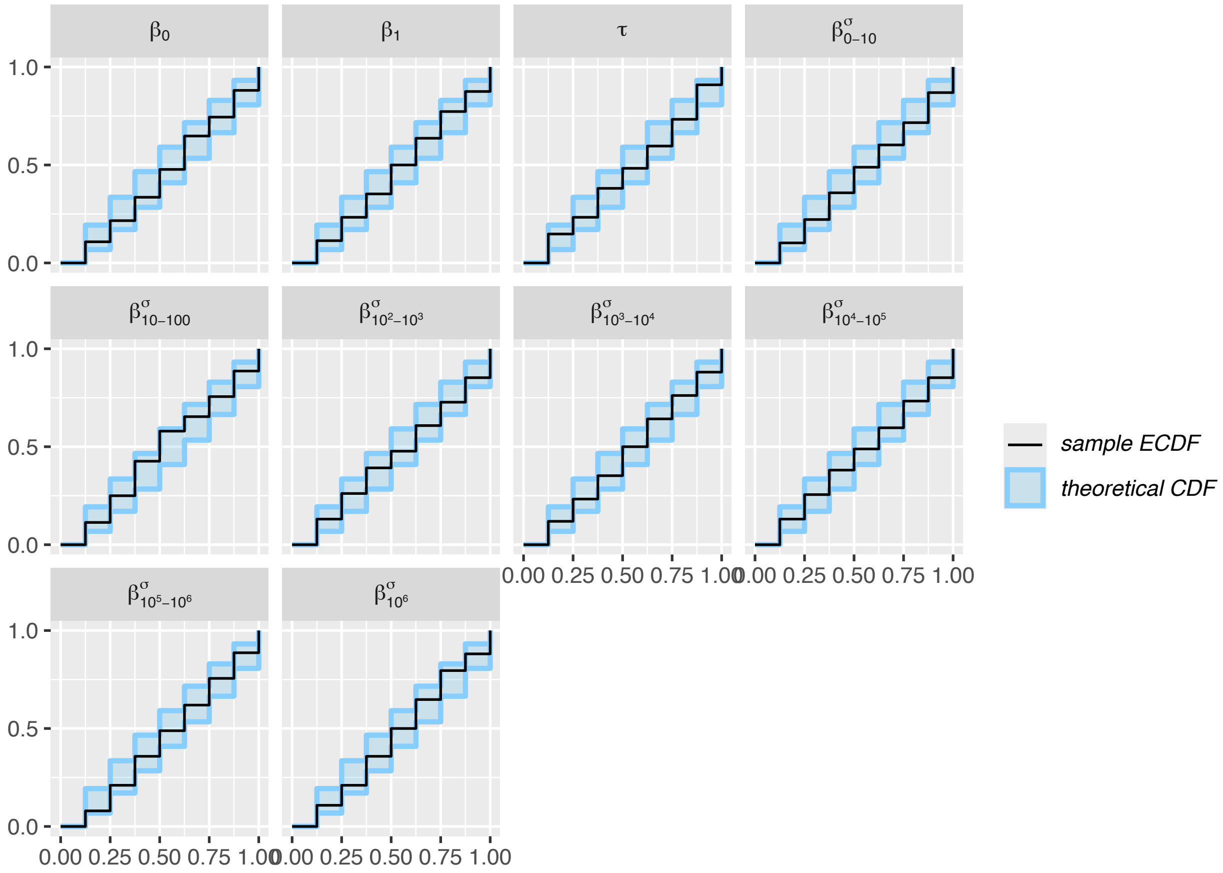

Figure S11: Model 1.3 had reasonable coverage for the parameters of interest. Results are shown for n = 176 simulated data sets. Blue line is 1:1 line, and shading shows 95% uncertainty interval for the coverage.

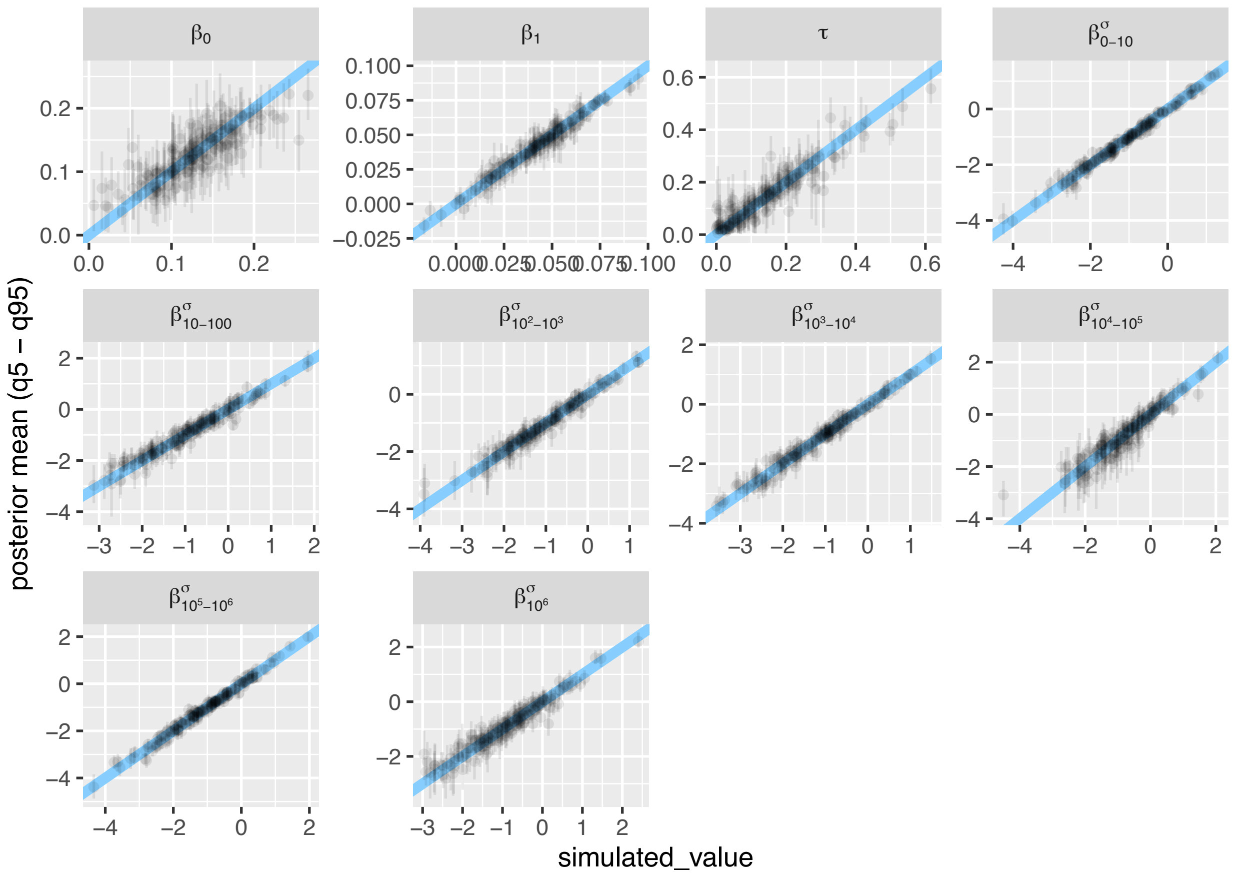

Figure S12: Model 1.3 was able to recover known parameter values, though with some uncertainty (most notably for β_0, and τ). Results are shown for n = 176 simulated data sets; "simulated_value" (x-axis) is the known value of the parameter for a given simulation. Each point shows a parameter estimate, whiskers show 95% credible interval; diagonal line is the 1:1 line.

Model 1.4

The next model examined in the main text included varying study-level residual variation:

$$z_{ij} \sim N(\mu_{ij}, s_{ij}^2 + \sigma_{ij}^2),$$

$$\mu_{ij} = \beta_0 + \beta_{0i} + \beta_1 X_i,$$

$$\beta_{0i} \sim N(0, \tau^2),$$

$$log(\sigma_{ij}) = \beta_0^{\sigma} + \beta_{0i}^{\sigma},$$

$$\beta_{0i}^{\sigma} \sim N(0, \zeta^2),$$

where β_0^σ is the average residual variation (on a log-scale), and β_{0i}^σ is a normally distributed study-level varying intercept with zero mean and ζ standard deviation. The model was fit to the empirical data with weakly regularizing parameters:

$$\beta_0 \sim N(0.3, 1),$$

$$\beta_1, \beta_0^\sigma \sim N(0, 1),$$

$$[\tau, \zeta] \sim N(0, 1). \qquad \text{(Model 1.4)}$$

Model 1.4 had good convergence (all Rhats < 1.01), and showed a reasonable fit to the empirical data (Appendix S2: Fig. S13).

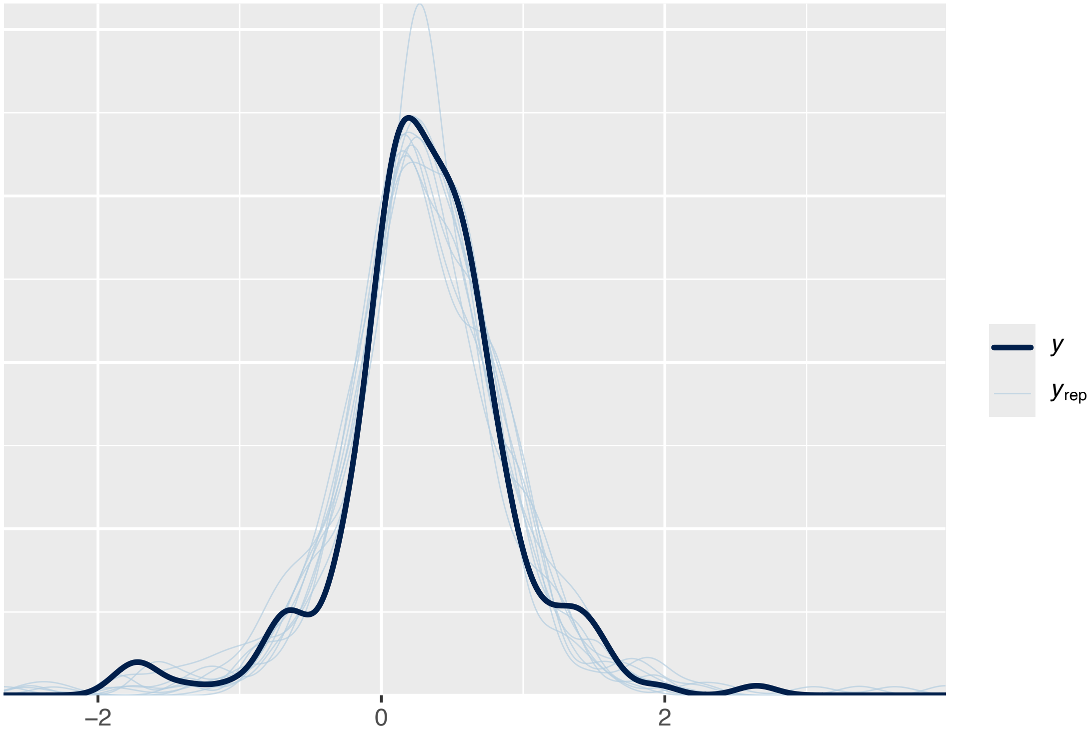

Figure S13: Posterior predictions from model 1.4 showed a good fit to the empirical data.

The parameter estimates from the fit of model 1.4 to empirical data (β_0: 0.17 [95% credible interval: 0.06 – 0.27]; β_1: 0.03 [95% credible interval: 0.02 – 0.05]; τ: 0.31 [95% credible interval: 0.22 – 0.40]; β_0^σ: -1.65 [95% credible interval: -2.15 – -1.26]; ζ: 0.89 [95% credible interval: 0.56 – 1.31]) were used to inform the following priors:

$$\beta_0 \sim N(0.17, 0.05),$$

$$\beta_1 \sim N(0.04, 0.02),$$

$$\tau \sim N(0, 0.2),$$

$$\beta_0^{\sigma} \sim N(-1.6, 0.25),$$

$$\zeta \sim N(0, 0.7),$$

which were combined with model 1.4 to simulate (fake) data sets.

Simulation-based calibration for model 1.4 showed that the rank statistics were approximately uniformly distributed (Appendix S2: Fig. S14), and that the coverage of parameters was reasonable (Appendix S2: Fig. S15). Finally, most of the known parameters were approximately recovered, though with a fair amount of uncertainty for the intercept (β_0), and particularly the varying intercept for study-level unexplained variation (ζ; Appendix S2: Fig. S16). The relatively poor (and highly uncertain) estimate of study-level unexplained variation (ζ) is likely due to the limited replication within studies (the median number of effect sizes per study was one; 74 of 101 studies had a single effect size).

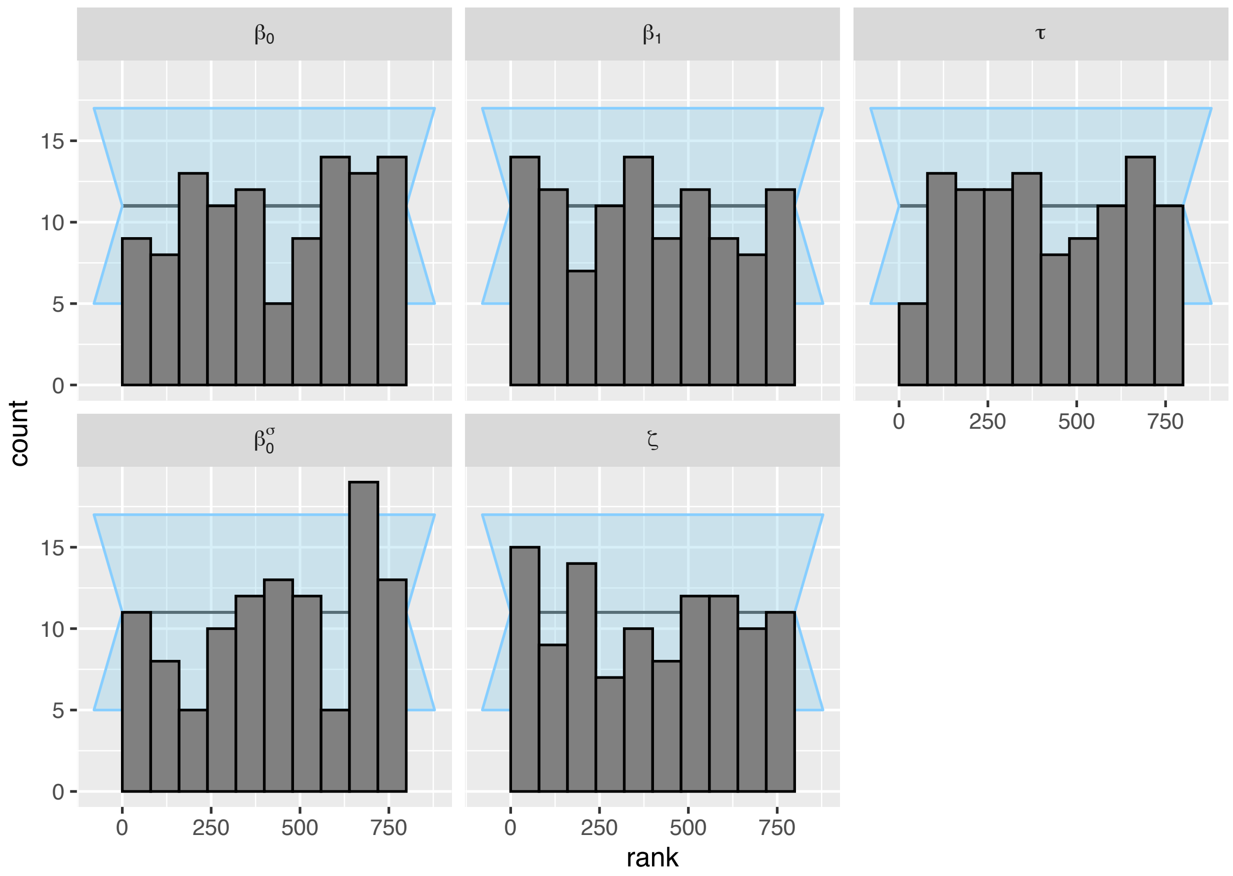

Figure S14: The posterior ranks of the prior draws were approximately normally distributed for the parameters of interest in model 1.4. Results are shown for n = 108 simulated data sets. Background (light blue shading) shows an approximate 95% interval for expected deviations.

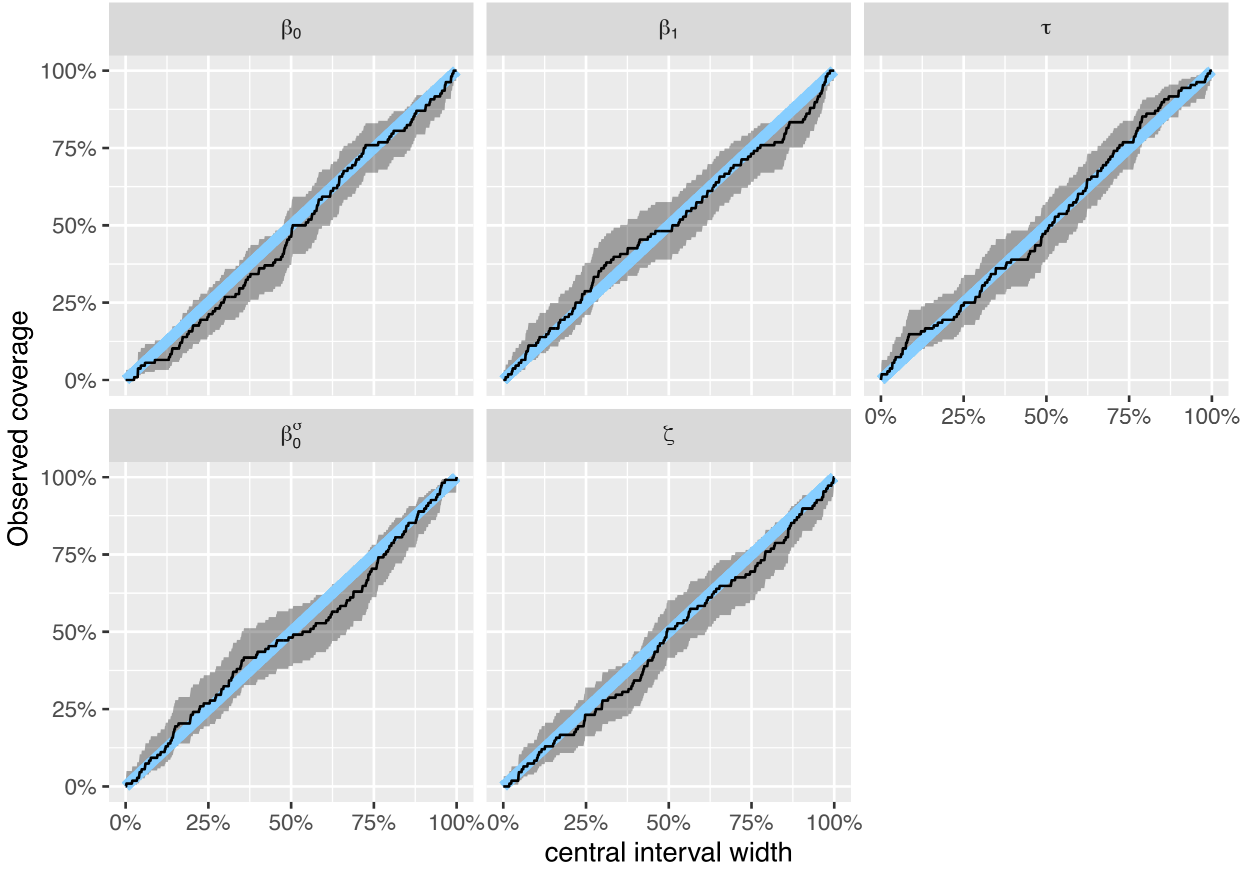

Figure S15: Model 1.4 had reasonable coverage for the parameters of interest, though the intercept (β_0) showed some discrepancies. Results are shown for n = 108 simulated data sets. Blue line is 1:1 line, and shading shows 95% uncertainty interval for the coverage.

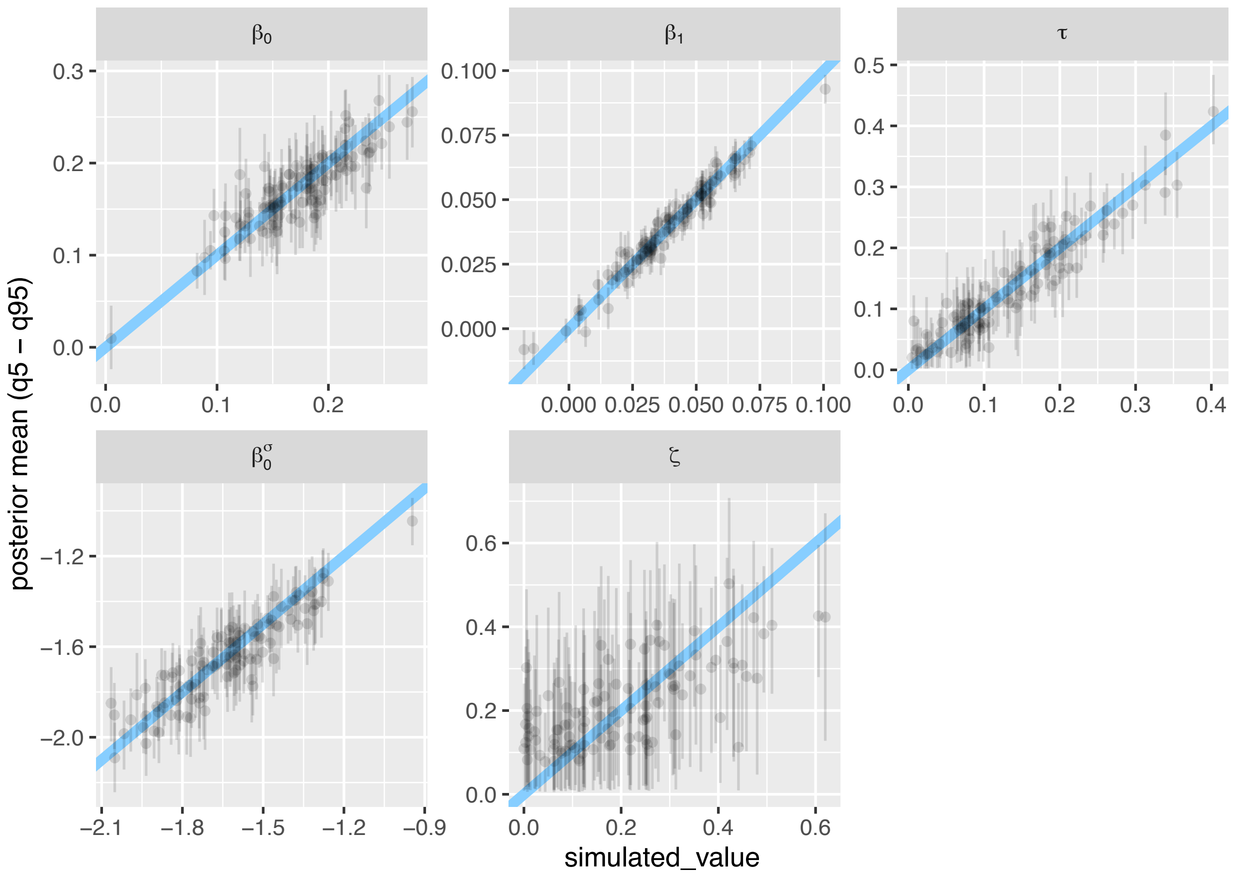

Figure S16: Model 1.4 was able to recover known parameter values, though with little precision for ζ. Results are shown for n = 108 simulated data sets; "simulated_value" (x-axis) is the known value of the parameter for a given simulation. Each point shows a parameter estimate, whiskers show 95% credible interval; diagonal line is the 1:1 line.

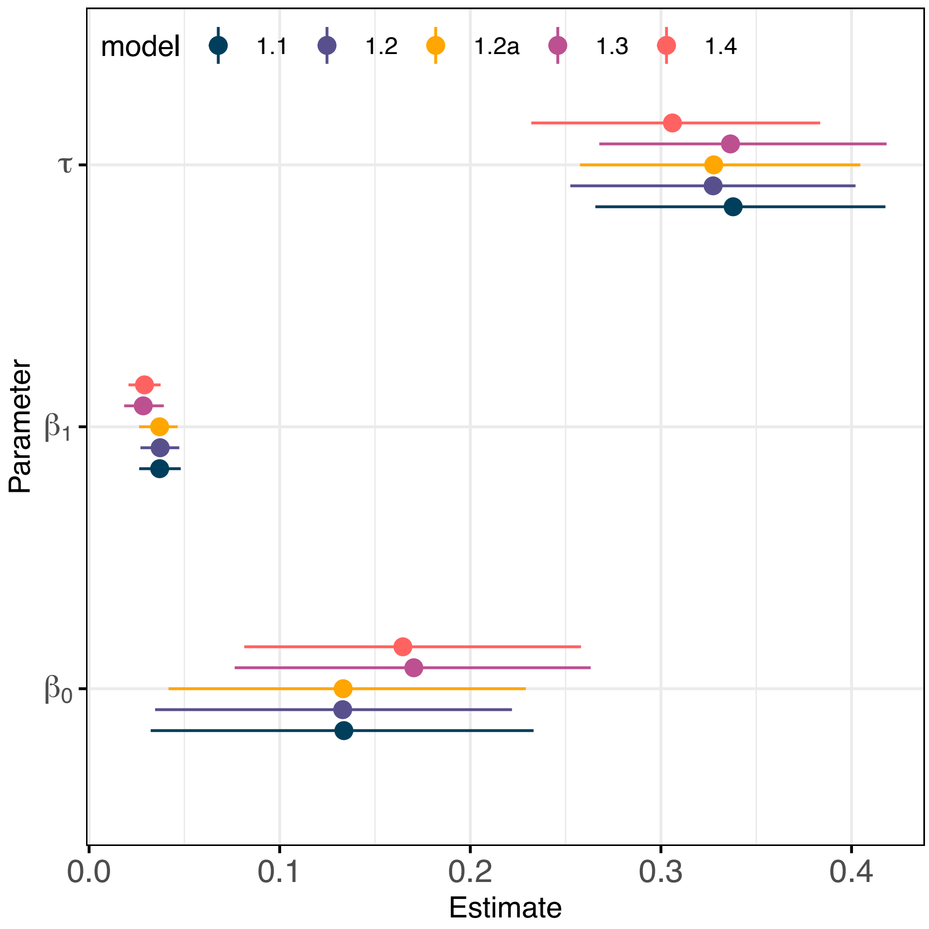

Figure S17: All models produced qualitatively similar estimates for shared parameters (β_0, β_1, τ).

References

Modrák, M., A. H. Moon, S. Kim, P. Bürkner, N. Huurre, K. Faltejsková, A. Gelman, and A. Vehtari. 2023. Simulation-based calibration checking for Bayesian computation: The choice of test quantities shapes sensitivity. Bayesian Analysis 1:1–28.

Peng, S., N. L. Kinlock, J. Gurevitch, and S. Peng. 2019. Correlation of native and exotic species richness: a global meta-analysis finds no invasion paradox across scales. Ecology 100:e02552.

Talts, S., M. Betancourt, D. Simpson, A. Vehtari, and A. Gelman. 2020, October 21. Validating Bayesian Inference Algorithms with Simulation-Based Calibration. arXiv.
