# Supplementary material for: Location‐scale models and cross validation to advance quantitative evidence synthesis
Source: Ecology. 2026 Jan 26;107(1):e70303. doi: 10.1002/ecy.70303 (PMC12834672; doi:10.1002/ecy.70303)
Supplement: Supplementary file 2 — Appendix S2. [file ECY-107-e70303-s001.pdf]

Appendix S2

Author: Shane Blowes

Title: Location-scale models and cross validation to advance quantitative evidence synthesis

Journal: Ecology

Case-study two: Simulation-based calibration and supplemental figures

I used Simulation-Based Calibration (Talts et al. 2020, Modrák et al. 2023) to check whether models could recover known parameter values with reasonable coverage (i.e., the probability that a constructed [e.g., credible] interval contains the true value), accuracy and precision. I focus calibration on the region of the parameter space around the empirical posterior (i.e., posterior simulation-based calibration; Säilynoja et al. 2025). Briefly, this involves: (1) fitting a model to the empirical data; (2) using the same model with priors informed by the fit to the empirical data to simulate many new (fake) datasets with the same size, shape and structure as the empirical data (i.e., the same number of observations, and the same number of groups for each level in the hierarchical structure); (3) refitting the model to each simulated data set; and, (4) calculating and plotting SBC diagnostics of model fits to simulated data to check for reasonable coverage (Talts et al. 2020, Modrák et al. 2023).

To examine model calibration, I focus on three plots from the SBC diagnostics: (1) a histogram of the posterior ranks of the prior draws, which (if the algorithm and model are working correctly) should be approximately normally distributed; (2) the empirical coverage of parameters of interest (coverage is the proportion of known variable values that fall within the interval: a well calibrated model would have coverage exactly matching the interval width, e.g., the 50% credible interval contains the known value 50% of the time); and, (3) a plot of estimated

parameter values as a function of known (simulated) parameter values for parameters of interest, which shows how accurately and precisely the model estimates focal parameters.

Model 2.1

To start case-study two I refit the model fit by Chase et al. (2020) to effort-standardized for species richness (S_{ij}) in fragment *j* from study *i*:

$$S_{ij} \sim lognormal(\mu_{ij}, \sigma^2)$$

$$\mu_{ij} = \beta_0 + \beta_{0i} + (\beta_1 + \beta_{1i})X_{ij},$$

$$[\beta_{0i}, \beta_{1i}]' \sim MVN(0, \boldsymbol{SRS}),$$

$$\boldsymbol{S} = \begin{bmatrix} \sigma_{0i} & 0 \\ 0 & \sigma_{1i} \end{bmatrix},$$

$$\boldsymbol{R} = \begin{bmatrix} 1 & \rho_{\sigma_{0i}\sigma_{1i}} \\ \rho_{\sigma_{0i}\sigma_{1i}} & 1 \end{bmatrix},$$

where X_{ij} is the fragment size on a (natural) log-scale, which was centered by subtracting the overall mean from each observation before modelling; β_{0i} and β_{1i} are study-level departures from the overall intercept and slope, respectively, drawn from a multivariate normal (*MVN*) distribution with standard deviations, σ_{0i} and σ_{1i}, that allowed for correlations ($\boldsymbol{R}$ matrix) between the varying intercepts and slopes. The model was fit to empirical data using weakly regularizing priors:

$$\beta_0 \sim N(2.5, 1),$$

$$\beta_1 \sim N(0, 1),$$

$$[\sigma_{0i}, \sigma_{1i}, \sigma] \sim N(0, 1),$$

$$\mathbf{R} \sim \text{LKJ}(1). \qquad \text{(Model 2.1)}$$

This model had good convergence (all Rhat <= 1.01), and showed a reasonable fit to the empirical data (Appendix S2: Fig. S1).

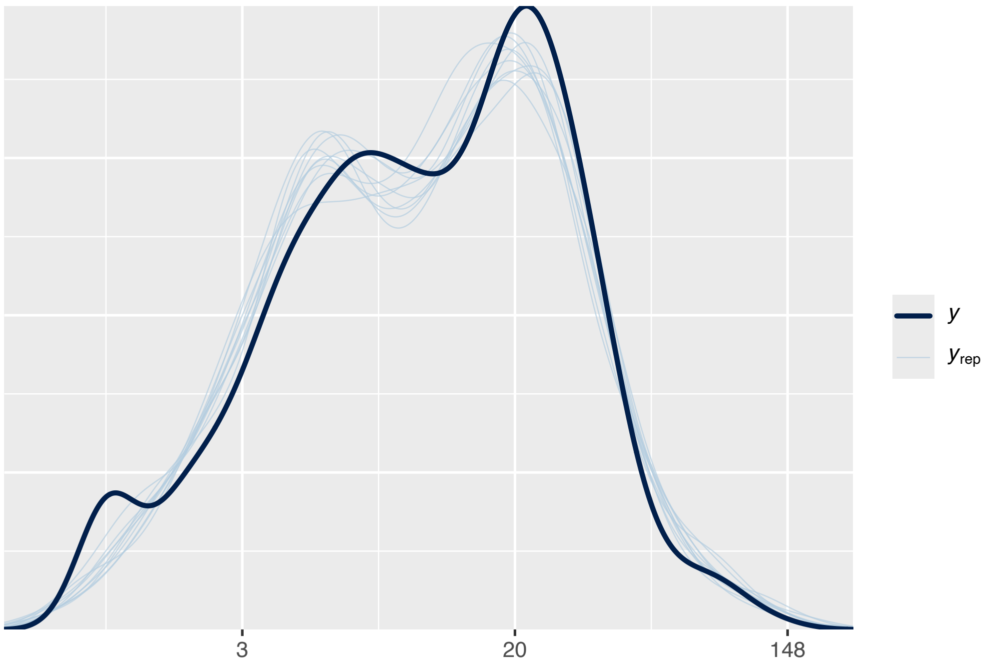

Figure S1: Posterior predictions from model 2.1 showed the model had a good ability to predict the observed data.

The parameter estimates from the fit of model 2.1 to empirical data (β_0: 2.46 [95% credible interval: 2.28 – 2.65]; β_1: 0.06 [95% credible interval: 0.04 – 0.07]; σ_{0i}: 1 [95% credible interval: 0.88 – 1.14]; σ_{1i}: 0.06 [95% credible interval: 0.04 – 0.08], and σ: 0.36 [95% credible interval: 0.34 – 0.37]) were used to inform the following priors:

$$\beta_0 \sim N(2.4, 0.1),$$

$$\beta_1 \sim N(0.06, 0.01),$$

$$\sigma_{0i} \sim N(1, 0.1),$$

$$\sigma_{1i} \sim N(0, 0.05),$$

$$\sigma \sim N(0.3, 0.05),$$

which were combined with model 2.1 to simulate (fake) data sets. To ensure as much realism as possible in the simulated data, each simulated data set retained the characteristics of the empirical data: 1509 observations distributed across 123 studies (with the same balance, i.e.,

fragment size distribution and fragments per study). I refit model S2.1 to each simulated data set, and identified models fit to fake data with poor diagnostics (e.g., divergent transitions and Rhats > 1.05); I report the number of simulated data sets used to calculate simulation diagnostics in figure captions.

Simulation-based calibration for model 2.1 showed that the rank statistics were approximately uniformly distributed (Appendix S2: Fig. S2), and that the coverage of parameters was reasonable (Appendix S2: Fig. S3); known parameters were recovered with reasonable accuracy (Appendix S2: Fig. S4). Note, (here and in subsequent calibrations for model extensions) I do not show parameters associated with the intercept (i.e., β_0, σ_{0i}) for the location component of the models, as these parameters are not readily interpretable. They correspond to the average richness (across all studies), and study-level variation around the overall average. Richness (and other diversity measures) are not readily comparable across this heterogeneous data compilation: richness in any given data set will depend on the sample grain and size of the species pool (among other things).

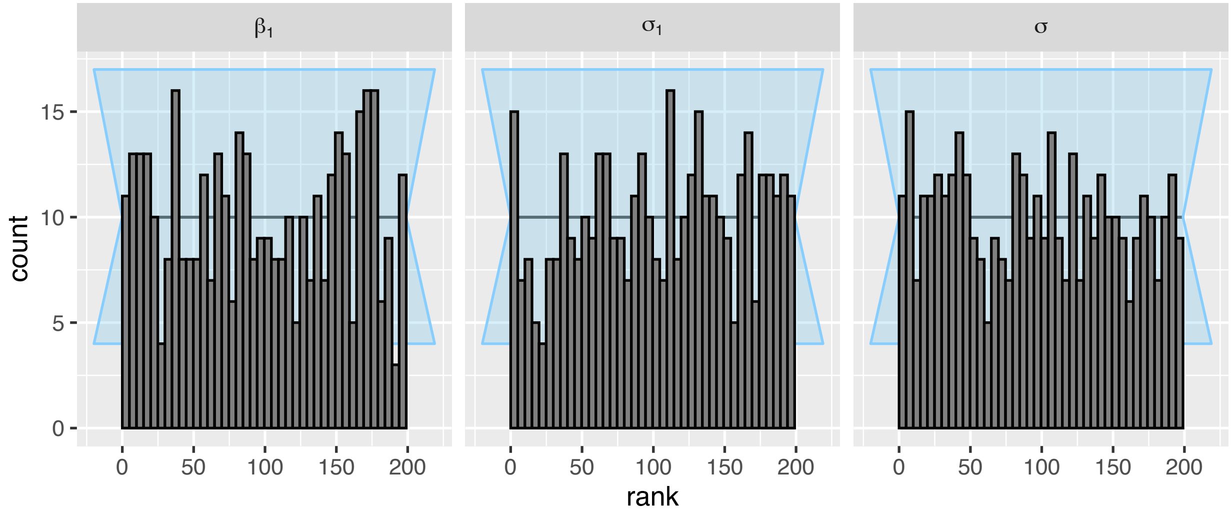

Figure S2: The posterior ranks of the prior draws were approximately normally distributed for the parameters of interest in model 2.1. Results are shown for n = 401 simulated data sets. Background (light blue shading) shows an approximate 95% interval for expected deviations.

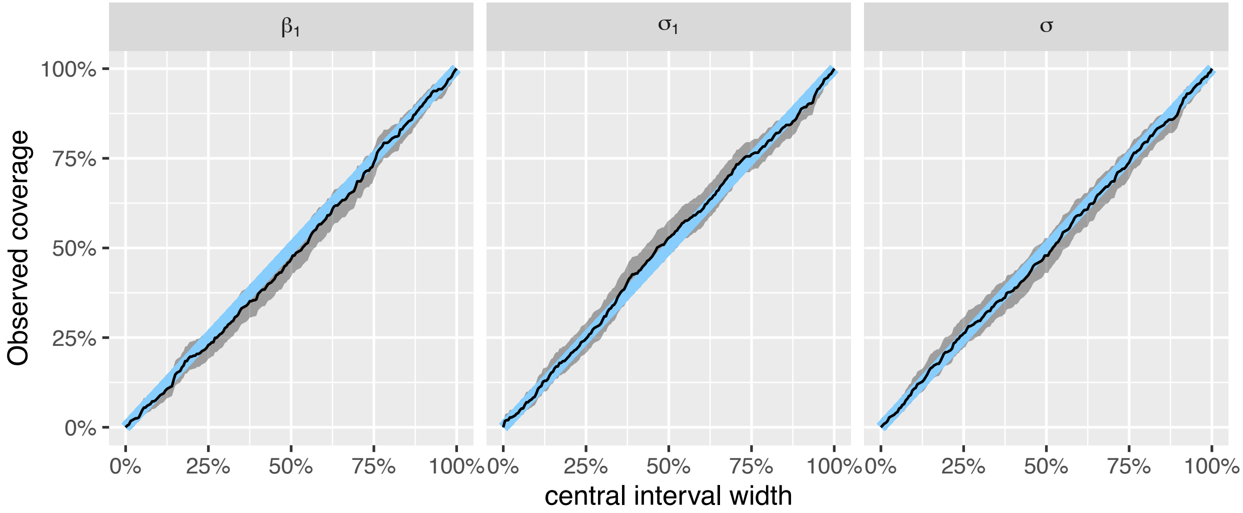

Figure S3: Model 2.1 had good coverage for the parameters of interest. Results are shown for n = 401 simulated data sets. Blue line is 1:1 line, and shading shows 95% uncertainty interval for the coverage.

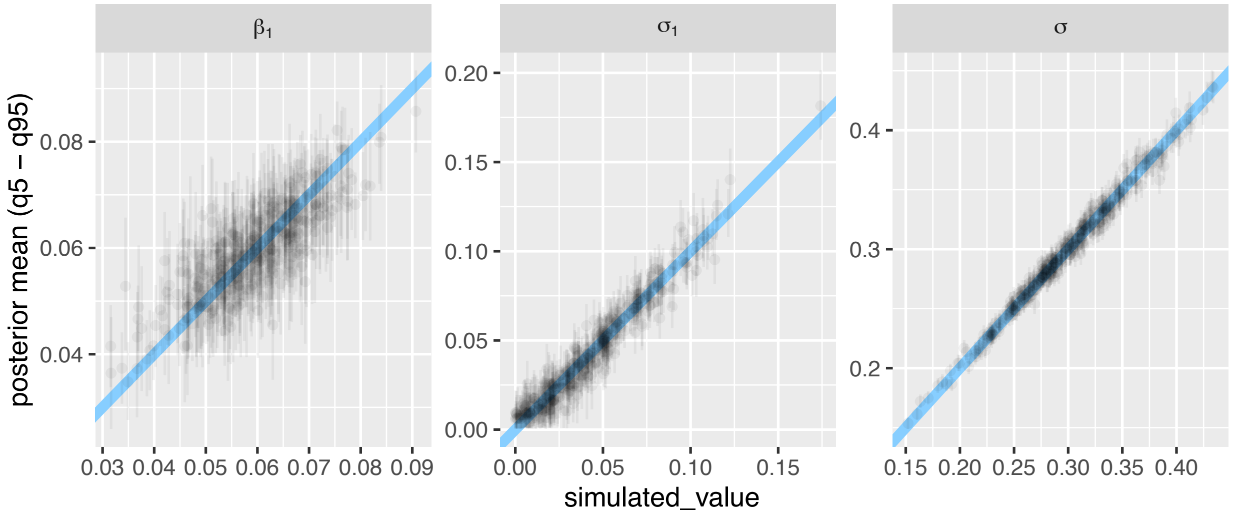

Figure S4: Model 2.1 was able to recover known parameter values, though with considerable uncertainty (with the exception of σ_1 and σ). Results are shown for n = 401 simulated data sets; "simulated_value" (x-axis) is the known value of the parameter for a given simulation. Each point shows a parameter estimate, whiskers show 95% credible interval; diagonal line is the 1:1 line.

Model 2.2

The first heteroscedastic model is motivated similarly to the varying intercepts and slopes per study, and allowed varying study-level residuals around an overall average residual standard

deviation. I estimated study-level residual variation independently of other varying study-level parameters for the mean:

$$S_{ij} \sim lognormal\left(\mu_{ij}, \sigma_i^2\right)$$

$$\mu_{ij} = \beta_0 + \beta_{0i} + (\beta_1 + \beta_{1i})X_{ij},$$

$$[\beta_{0i}, \beta_{1i}]' \sim MVN(0, \boldsymbol{SRS}),$$

$$\boldsymbol{S} = \begin{bmatrix} \sigma_{0i} & 0 \\ 0 & \sigma_{1i} \end{bmatrix},$$

$$\boldsymbol{R} = \begin{bmatrix} 1 & \rho_{\sigma_{0i}\sigma_{1i}} \\ \rho_{\sigma_{0i}\sigma_{1i}} & 1 \end{bmatrix},$$

$$log(\sigma_i) = \beta_0^\sigma + \beta_{0i}^\sigma,$$

$$\beta_{0i}^\sigma \sim N(0, \sigma_{0i}^\sigma),$$

where β_0^σ is the overall average of residual standard variation (on a log-scale), and β_{0i}^σ are the varying study-level departures (for the residual variation) drawn from a normal distribution with zero mean and standard deviation σ_{0i}^σ. I fit the model with weakly regularizing priors:

$$\beta_0 \sim N(2.5, 1),$$

$$\beta_1, \beta_0^\sigma \sim N(0, 1),$$

$$[\sigma_{0i}, \sigma_{1i}, \sigma_{0i}^\sigma] \sim N(0, 1),$$

$$\mathbf{R} \sim LKJ(1). \qquad \text{(Model 2.2)}$$

This model had good convergence (all Rhat <= 1.01), and showed a reasonable fit to the empirical data (Appendix S2: Fig. S5).

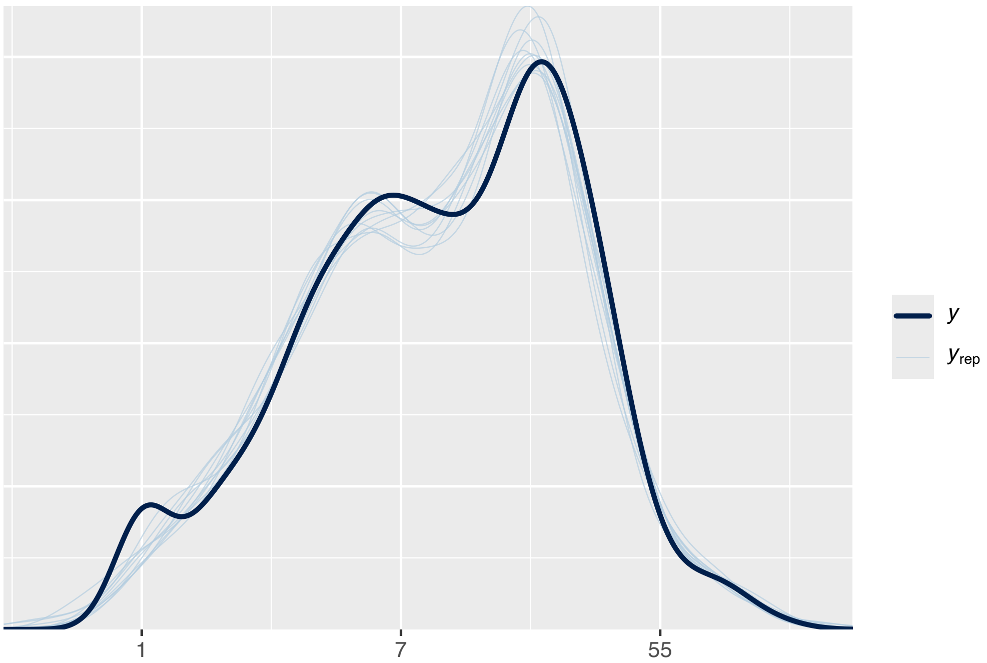

Figure S5: Posterior predictions from model 2.2 showed the model had a good ability to predict the observed data.

The parameter estimates from the fit of model 2.2 to empirical data (β_0: 2.48 [95% credible interval: 2.31 – 2.66]; β_1: 0.05 [95% credible interval: 0.04 – 0.07]; σ_{0i}: 1 [95% credible interval: 0.88 – 1.15]; σ_{1i}: 0.06 [95% credible interval: 0.04 – 0.07], β_0^{σ}: -1.23 [95% credible interval: -1.32 – -1.13], and σ_{0i}^{σ}: 0.44 [95% credible interval: 0.36 – 0.53]) were used to inform the following priors:

$$\beta_0 \sim N(2.5, 0.1),$$

$$\beta_1 \sim N(0.05, 0.01),$$

$$\sigma_{0i} \sim N(1, 0.1),$$

$$\sigma_{1i} \sim N(0.05, 0.05),$$

$$\beta_0^{\sigma} \sim N(-1.2, 0.1),$$

$$\sigma_{0i}^{\sigma} \sim N(0.4, 0.1).$$

Simulation-based calibration for model 2.2 showed that the rank statistics were approximately uniformly distributed (Appendix S2: Fig. S6), and that the coverage of parameters was reasonable (Appendix S2: Fig. S7); known parameters were approximately recovered (Appendix S2: Fig. S8).

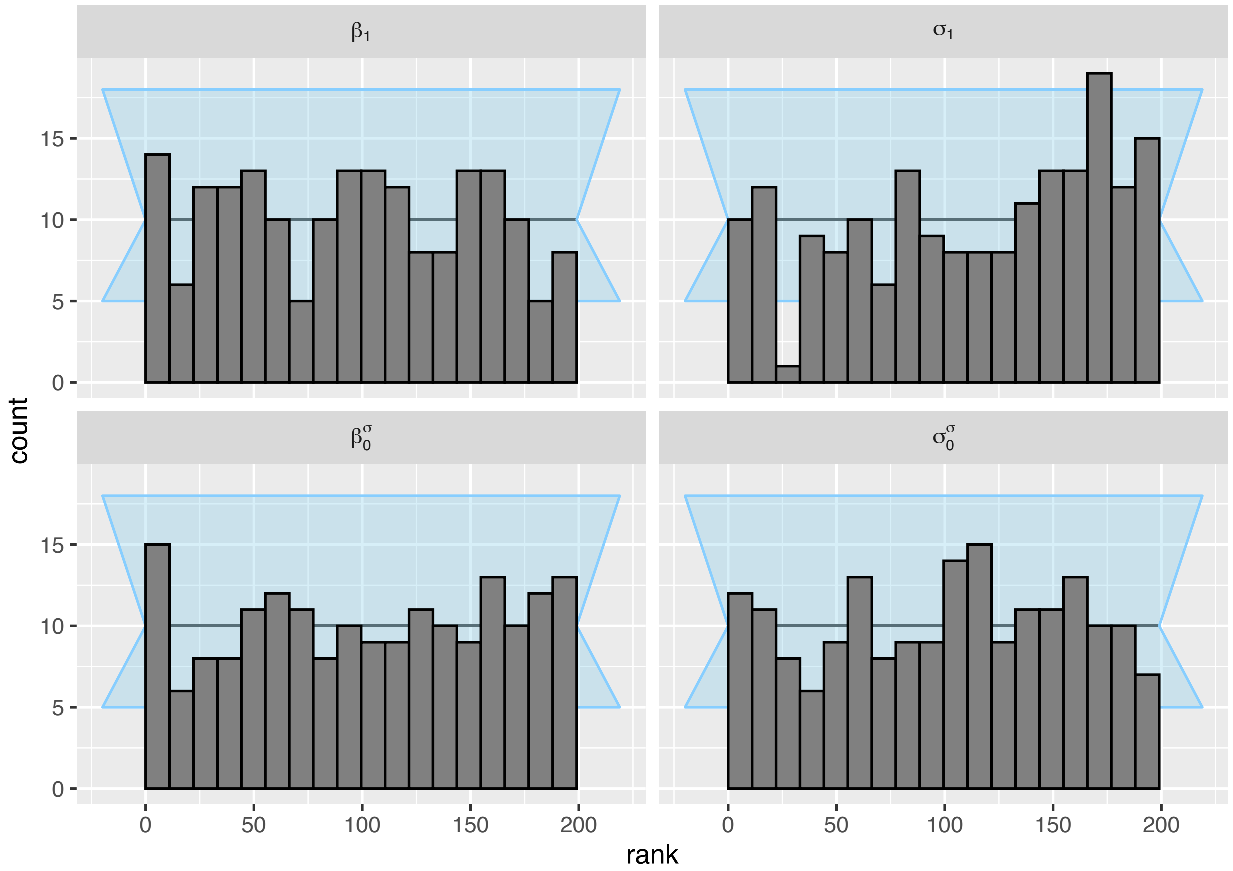

Figure S6: The posterior ranks of the prior draws were approximately normally distributed for the parameters of interest in model 2.2. Results are shown for n = 185 simulated data sets. Background (light blue shading) shows an approximate 95% interval for expected deviations.

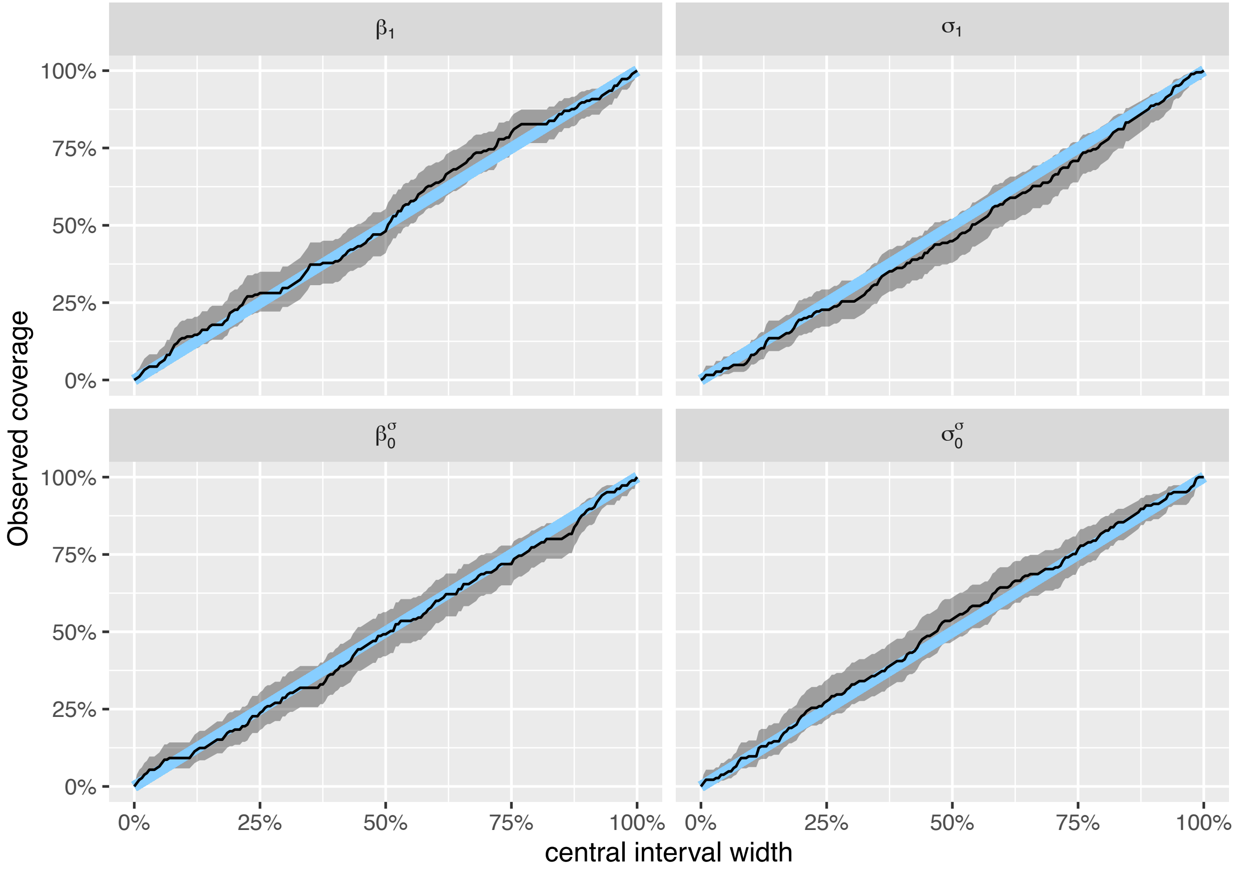

Figure S7: Model 2.2 had good coverage for the parameters of interest. Results are shown for n = 185 simulated data sets. Blue line is 1:1 line, and shading shows 95% uncertainty interval for the coverage.

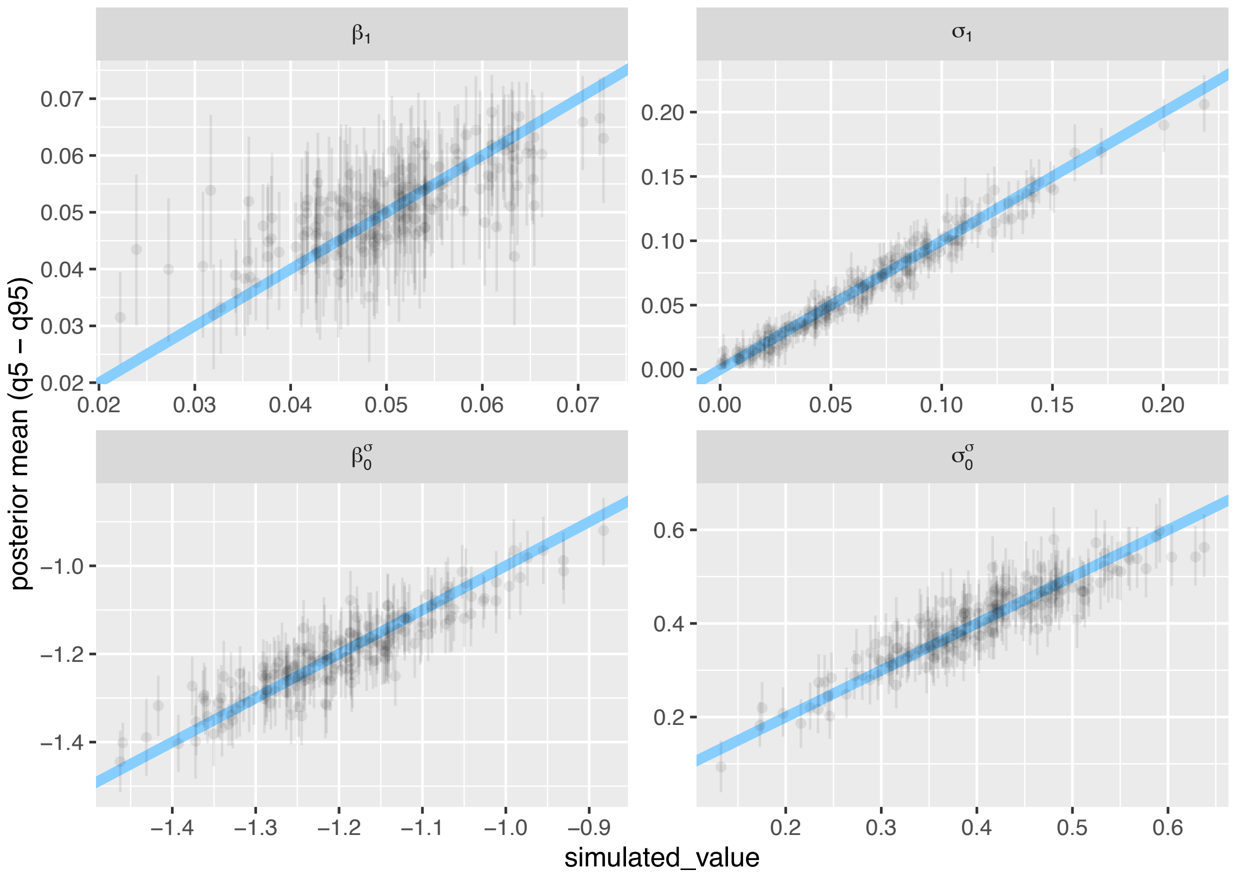

Figure S8: Model 2.2 was able to recover known parameter values fairly accurately, though with relatively low precision (with the exception of σ_1). Results are shown for n = 185 simulated data sets; "simulated_value" (x-axis) is the known value of the parameter for a given simulation. Each point shows a parameter estimate, whiskers show 95% credible interval; diagonal line is the 1:1 line.

Model 2.3

Next, I extend this model to include (log) fragment size as a predictor of residual variation, and again allow it to varying between studies independently of varying study-level parameters for the mean:

$$S_{ij} \sim lognormal\left(\mu_{ij}, \sigma_i^2\right)$$

$$\mu_{ij} = \beta_0 + \beta_{0i} + (\beta_1 + \beta_{1i})X_{ij},$$

$$[\beta_{0i}, \beta_{1i}]' \sim MVN(0, \boldsymbol{SRS}),$$

$$\boldsymbol{S} = \begin{bmatrix} \sigma_{0i} & 0 \\ 0 & \sigma_{1i} \end{bmatrix},$$

$$\boldsymbol{R} = \begin{bmatrix} 1 & \rho_{\sigma_{0i}\sigma_{1i}} \\ \rho_{\sigma_{0i}\sigma_{1i}} & 1 \end{bmatrix},$$

$$log(\sigma_i) = \beta_0^\sigma + \beta_{0i}^\sigma + (\beta_1^\sigma + \beta_{1i}^\sigma)X_i,$$

$$[\beta_{0i}^\sigma, \beta_{1i}^\sigma]' \sim MVN(0, \boldsymbol{S^\sigma R^\sigma S^\sigma}),$$

$$\boldsymbol{S^\sigma} = \begin{bmatrix} \sigma_{0i}^\sigma & 0 \\ 0 & \sigma_{1i}^\sigma \end{bmatrix},$$

$$\boldsymbol{R^\sigma} = \begin{bmatrix} 1 & \rho_{\sigma_{0i}^\sigma\sigma_{1i}^\sigma} \\ \rho_{\sigma_{0i}^\sigma\sigma_{1i}^\sigma} & 1 \end{bmatrix},$$

where β_0^σ is the overall average residual variation, and β_1^σ is the overall average slope of residual variation with fragment size; β_{0i}^σ and β_{1i}^σ are the varying study-level departures from the intercept and slope, respectively, and were drawn from a multivariate normal distribution with zero mean and standard deviation σ_{0i}^σ and σ_{1i}^σ, with correlations estimated in matrix $\boldsymbol{R^\sigma}$. I fit the model with weakly regularizing priors:

$$\beta_0 \sim N(2.5, 1),$$

$$\beta_1, \beta_0^\sigma, \beta_1^\sigma \sim N(0, 1),$$

$$[\sigma_{0i}, \sigma_{1i}] \sim N(0, 1),$$

$$\mathbf{R} \sim LKJ(1),$$

$$[\sigma_{0i}^\sigma, \sigma_{1i}^\sigma] \sim N(0,1),$$

$$\boldsymbol{R^\sigma} \sim \text{LKJ}(1). \qquad \text{(Model 2.3)}$$

This model had good convergence (all Rhat <= 1.02), and showed a reasonable fit to the empirical data (Appendix S2: Fig. S9).

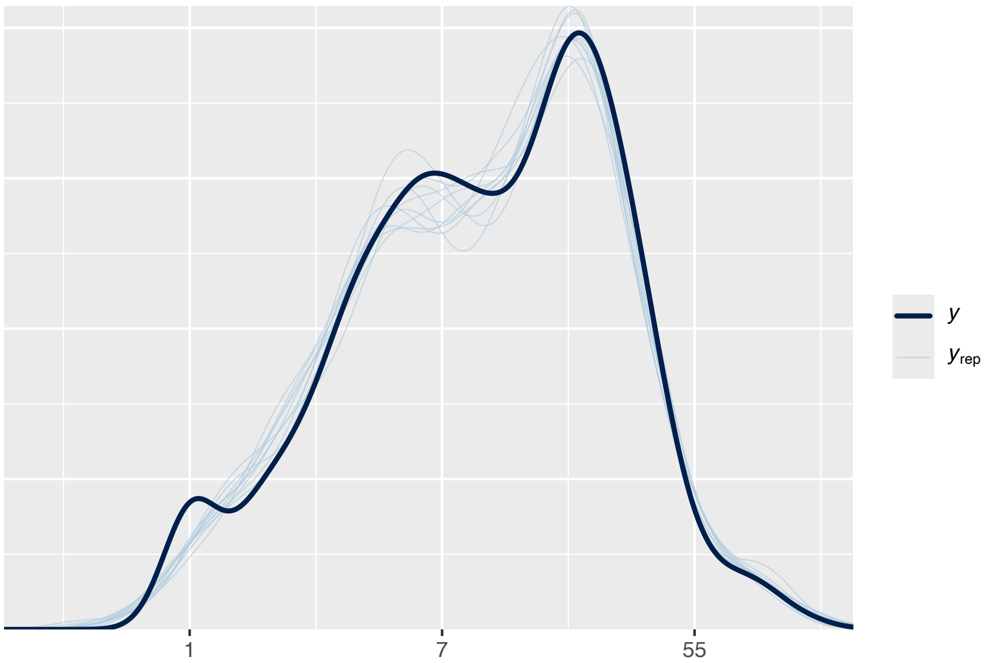

Figure S9: Posterior predictions from model 2.3 showed good fidelity to the observed data.

The parameter estimates from the fit of model 2.3 to empirical data (β_0: 2.48 [95% credible interval: 2.3 – 2.66]; β_1: 0.05 [95% credible interval: 0.03 – 0.06]; σ_{0i}: 1.01 [95% credible interval: 0.89 – 1.14]; σ_{1i}: 0.06 [95% credible interval: 0.04 – 0.07]; β_0^σ: -1.25 [95% credible interval: -1.35 – -1.15]; β_1^σ: -0.06 [95% credible interval: -0.09 – -0.03]; σ_{0i}^σ: 0.45 [95% credible interval: 0.37 – 0.55], and σ_{1i}^σ: 0.06 [95% credible interval: 0.01 – 0.1]) were used to inform the following priors:

$$\beta_0 \sim N(2.5, 0.1),$$

$$\beta_1 \sim N(0.05, 0.01),$$

$$\sigma_{0i} \sim N(1, 0.1),$$

$$\sigma_{1i} \sim N(0.05, 0.05),$$

$$\beta_0^\sigma \sim N(-1.2, 0.1),$$

$$\sigma_{0i}^\sigma \sim N(0.4, 0.1).$$

Simulation-based calibration for model 2.3 showed that the rank statistics were approximately uniformly distributed (Appendix S2: Fig. S10), and that the coverage of parameters was reasonable (Appendix S2: Fig. S11); known parameters were approximately recovered (Appendix S2: Fig. S12).

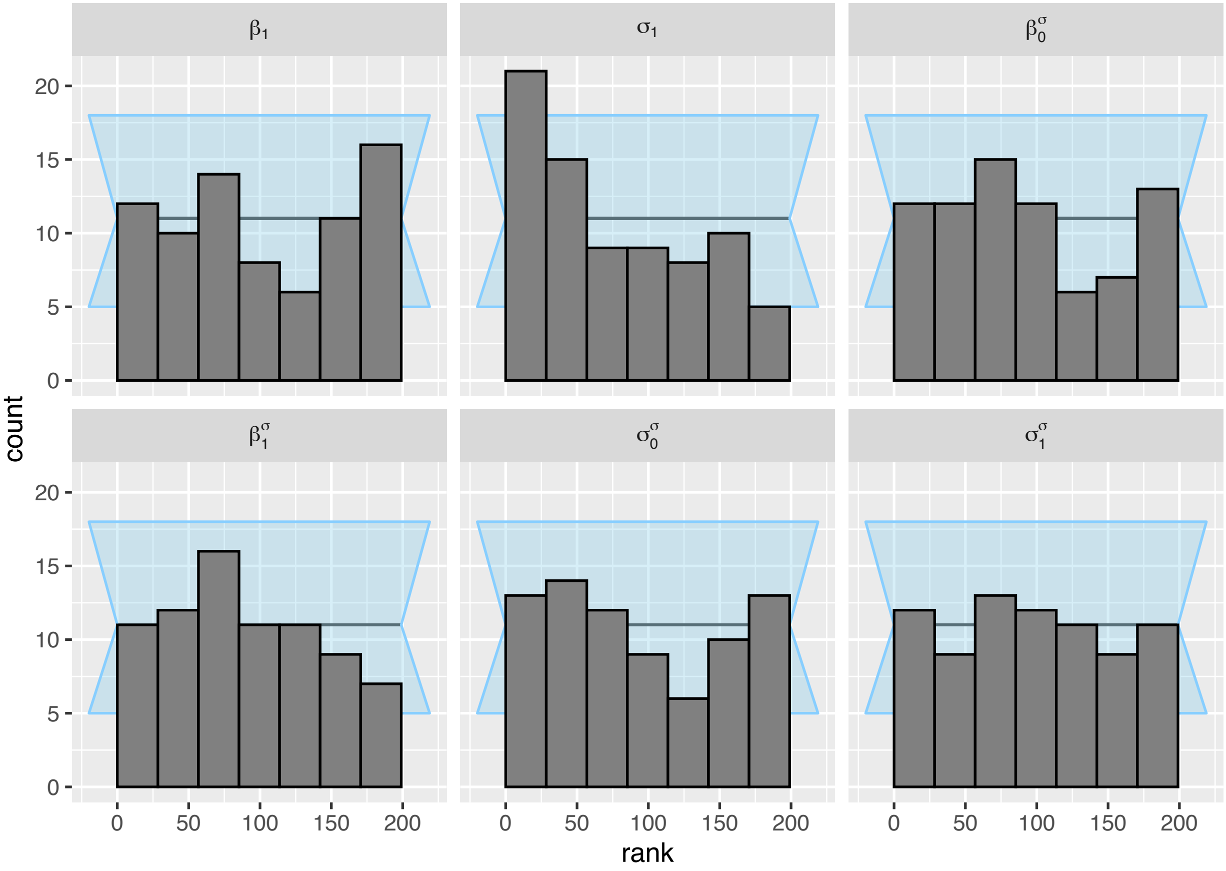

Figure S10: The posterior ranks of the prior draws were approximately normally distributed for the parameters of interest in model 2.3. Results are shown for n = 77 simulated data sets. Background (light blue shading) shows an approximate 95% interval for expected deviations.

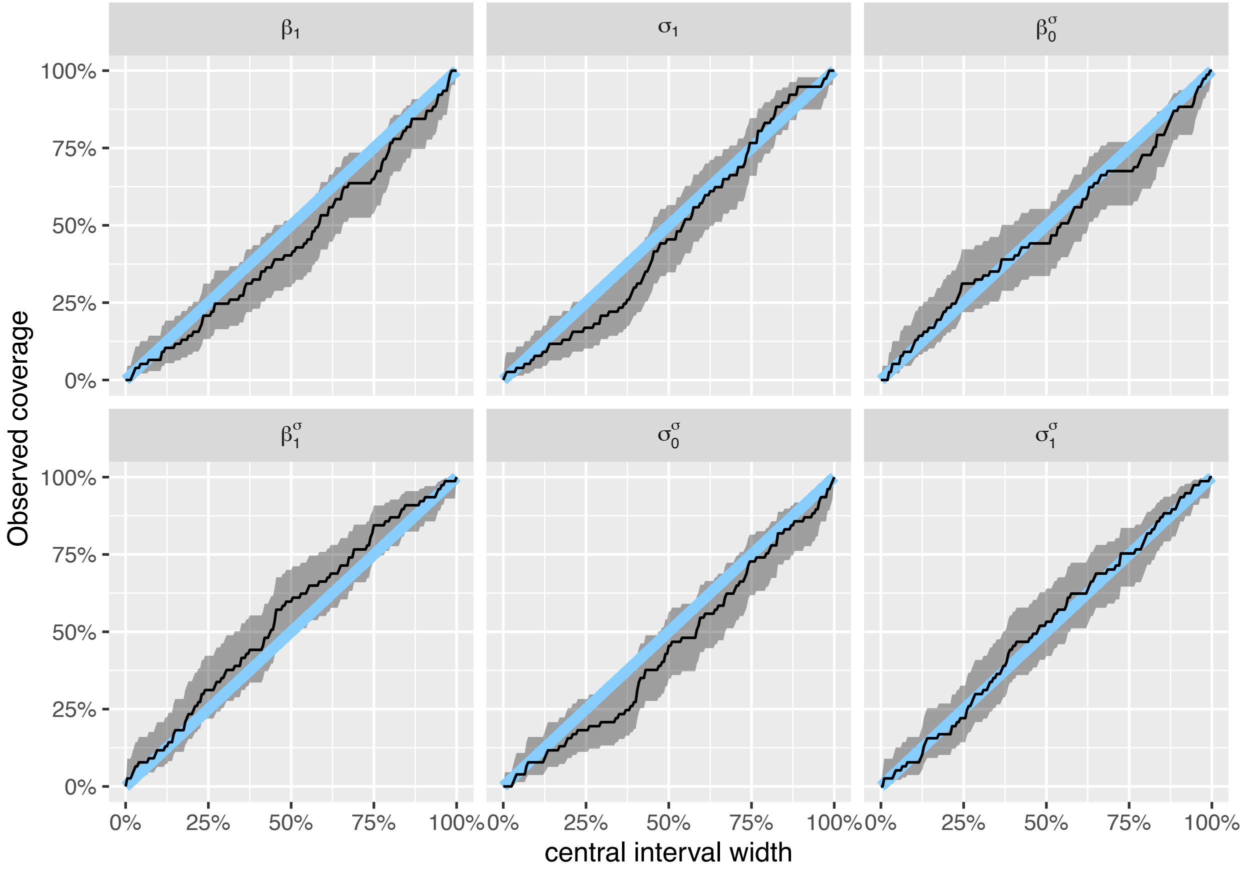

Figure S11: Model 2.3 had good coverage for all parameters of interest. Results are shown for n = 77 simulated data sets. Blue line is 1:1 line, and shading shows 95% uncertainty interval for the coverage.

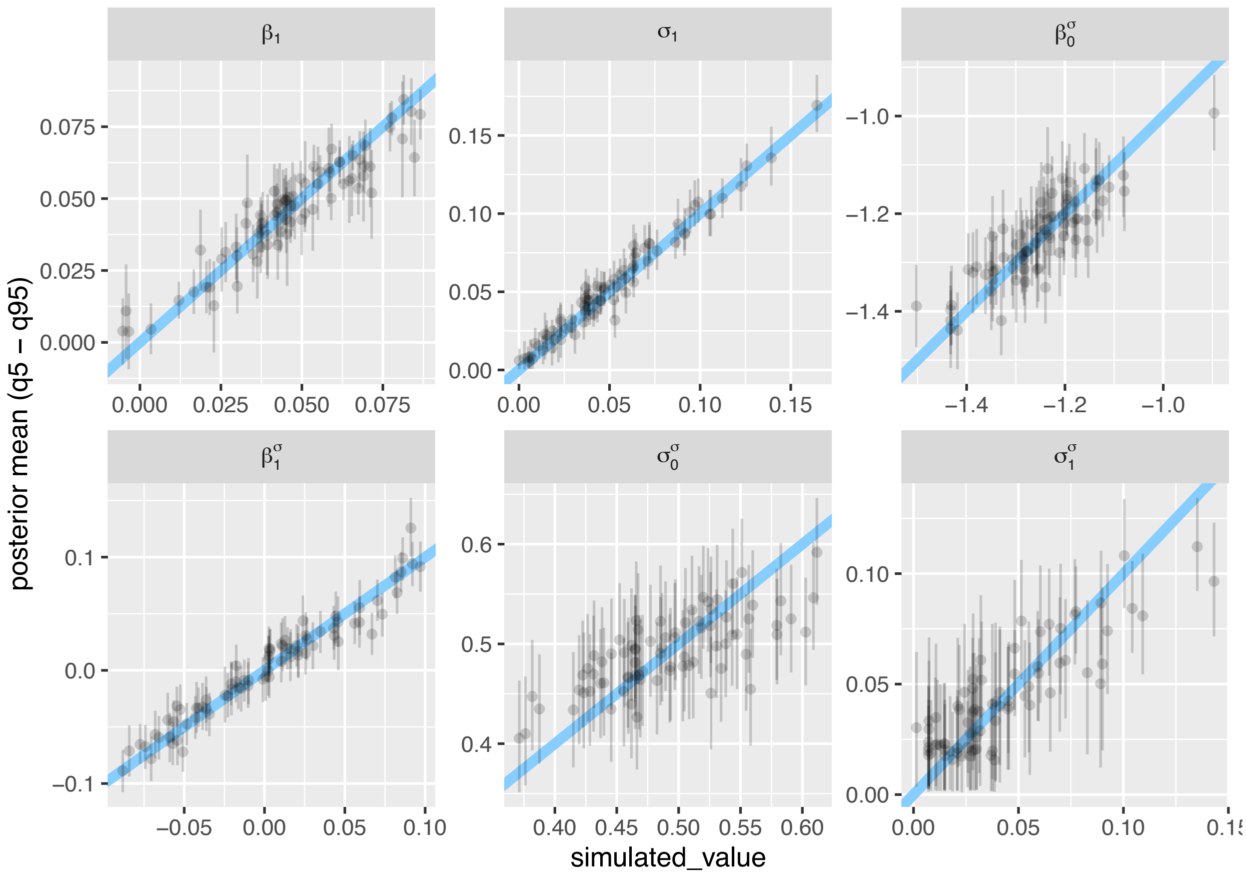

Figure S12: Model 2.3 was able to recover most parameters with reasonable accuracy, albeit with low precision (especially for σ_0^σ and σ_1^σ). Results are shown for n = 77 simulated data sets; "simulated_value" (x-axis) is the known value of the parameter for a given simulation. Each point shows a parameter estimate, whiskers show 95% credible interval; diagonal line is the 1:1 line.

Model 2.4

The next model is the same as model 2.2 (i.e., varying study-level residuals), but allows the study-level residuals to covary with the other varying parameters for the mean:

$$S_{ij} \sim lognormal\left(\mu_{ij}, \sigma_i^2\right)$$

$$\mu_{ij} = \beta_0 + \beta_{0i} + (\beta_1 + \beta_{1i})X_{ij},$$

$$log(\sigma_i) = \beta_0^\sigma + \beta_{0i}^\sigma,$$

$$[\beta_{0i}, \beta_{1i}, \beta_{0i}^\sigma]' \sim MVN(0, \boldsymbol{SRS}),$$

$$\boldsymbol{S} = \begin{bmatrix} \sigma_{0i} & 0 & 0 \\ 0 & \sigma_{1i} & 0 \\ 0 & 0 & \sigma_{0i}^{\sigma} \end{bmatrix},$$

$$\boldsymbol{R} = \begin{bmatrix} 1 & \rho_{\sigma_{0i}\sigma_{1i}} & \rho_{\sigma_{0i}\sigma_{0i}^{\sigma}} \\ \rho_{\sigma_{0i}\sigma_{1i}} & 1 & \rho_{\sigma_{1i}\sigma_{0i}^{\sigma}} \\ \rho_{\sigma_{0i}\sigma_{0i}^{\sigma}} & \rho_{\sigma_{1i}\sigma_{0i}^{\sigma}} & 1 \end{bmatrix}.$$

The model was fit to empirical data with weakly regularizing priors:

$$\beta_0 \sim N(2.5, 1),$$

$$\beta_1, \beta_0^{\sigma} \sim N(0, 1),$$

$$[\sigma_{0i}, \sigma_{1i}, \sigma_{0i}^{\sigma}] \sim N(0, 1),$$

$$\mathbf{R} \sim LKJ(1). \qquad \text{(Model 2.4)}$$

This model had good convergence (all Rhat <= 1.02), and showed a reasonable fit to the empirical data (Appendix S2: Fig. S13).

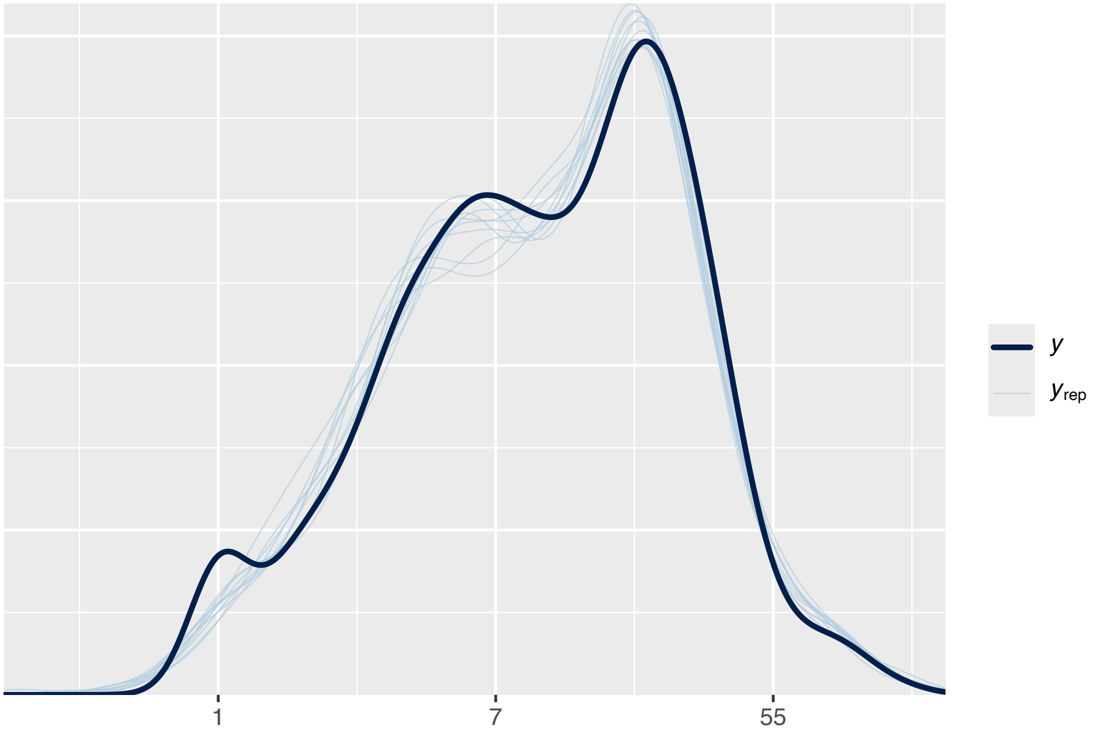

Figure S13: Posterior predictions showed that model 2.4 was able to make predict the observed data well.

The parameter estimates from the fit of model 2.4 to empirical data (β_0: 2.46 [95% credible interval: 2.29 – 2.66]; β_1: 0.05 [95% credible interval: 0.04 – 0.07]; σ_{0i}: 1 [95% credible interval: 0.89 – 1.13]; σ_{1i}: 0.06 [95% credible interval: 0.04 – 0.07], β_0^{σ}: -1.24 [95% credible interval: -1.35 – -1.15], and σ_{0i}^{σ}: 0.44 [95% credible interval: 0.37 – 0.53]) were used to inform the following priors:

$$\beta_0 \sim N(2.5,0.1),$$

$$\beta_1 \sim N(0.05, 0.01),$$

$$\sigma_{0i} \sim N(1, 0.1),$$

$$\sigma_{1i} \sim N(0.05, 0.05),$$

$$\beta_0^{\sigma} \sim N(-1.2, 0.1),$$

$$\sigma_{0i}^{\sigma} \sim N(0.4,0.1).$$

Simulation-based calibration for model 2.4 showed that the rank statistics were approximately uniformly distributed (Appendix S2: Fig. S14), and that the coverage of parameters was reasonable (Appendix S2: Fig. S15); known parameters were approximately recovered (Appendix S2: Fig. S16).

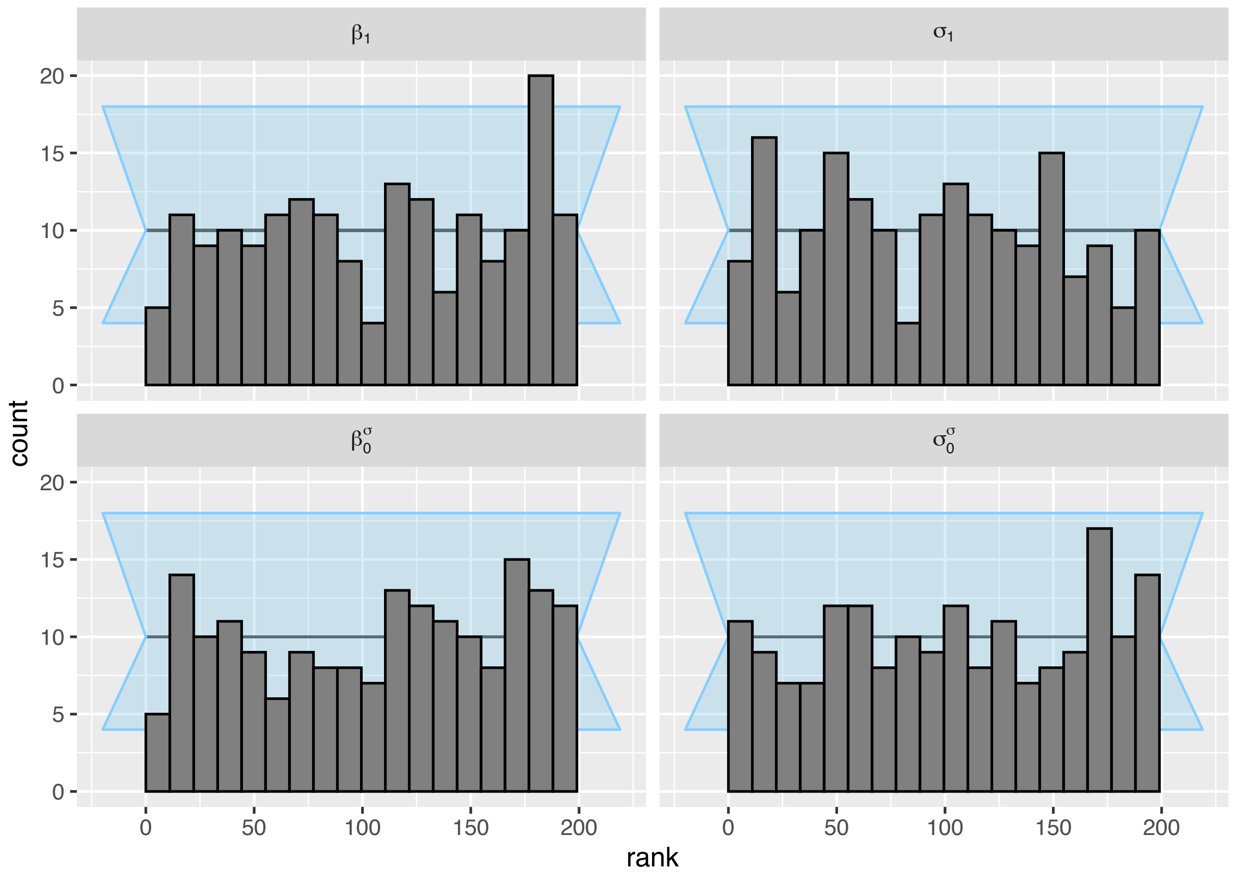

Figure S14: The posterior ranks of the prior draws were approximately normally distributed for the parameters of interest in model 2.4. Results are shown for n = 181 simulated data sets. Background (light blue shading) shows an approximate 95% interval for expected deviations.

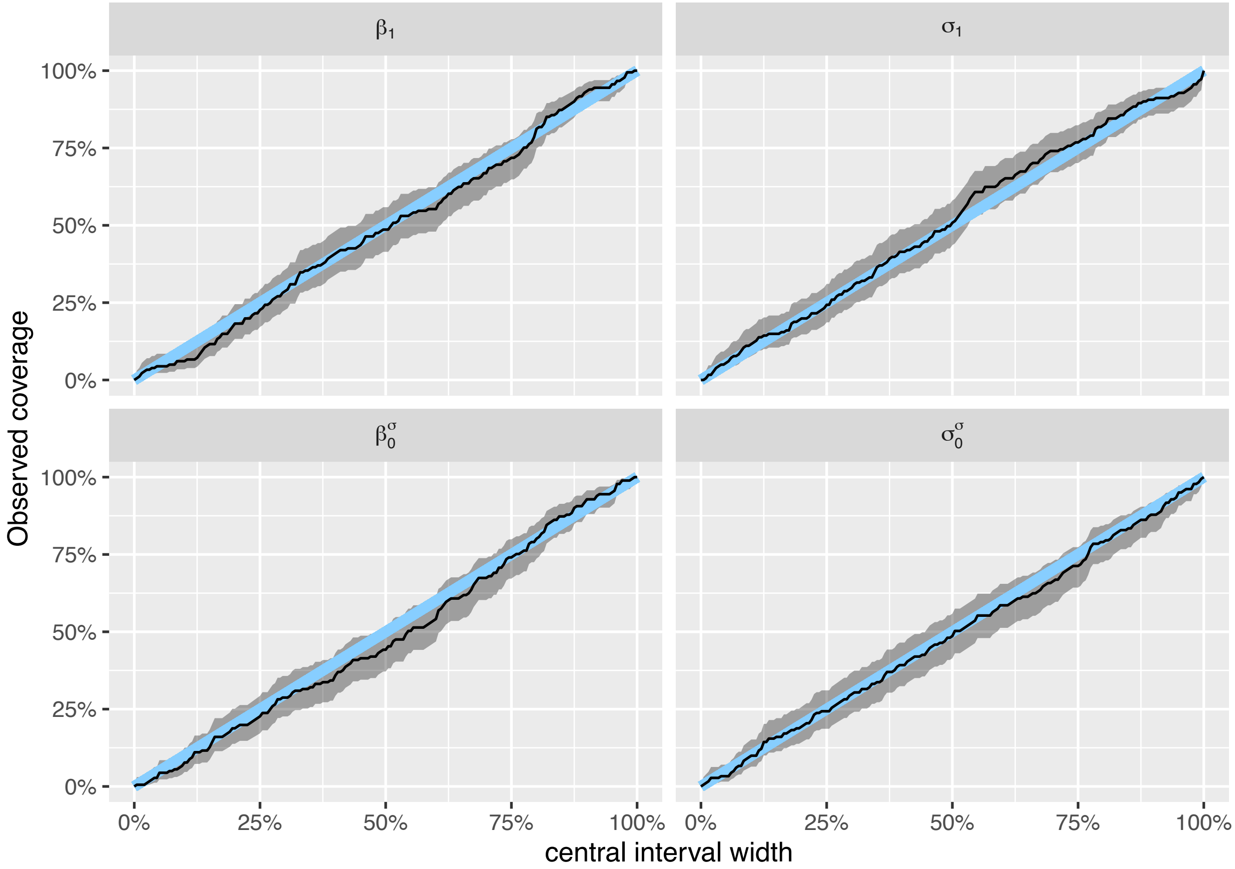

Figure S15: Model 2.4 had good coverage for all parameters of interest. Results are shown for n = 181 simulated data sets. Blue line is 1:1 line, and shading shows 95% uncertainty interval for the coverage.

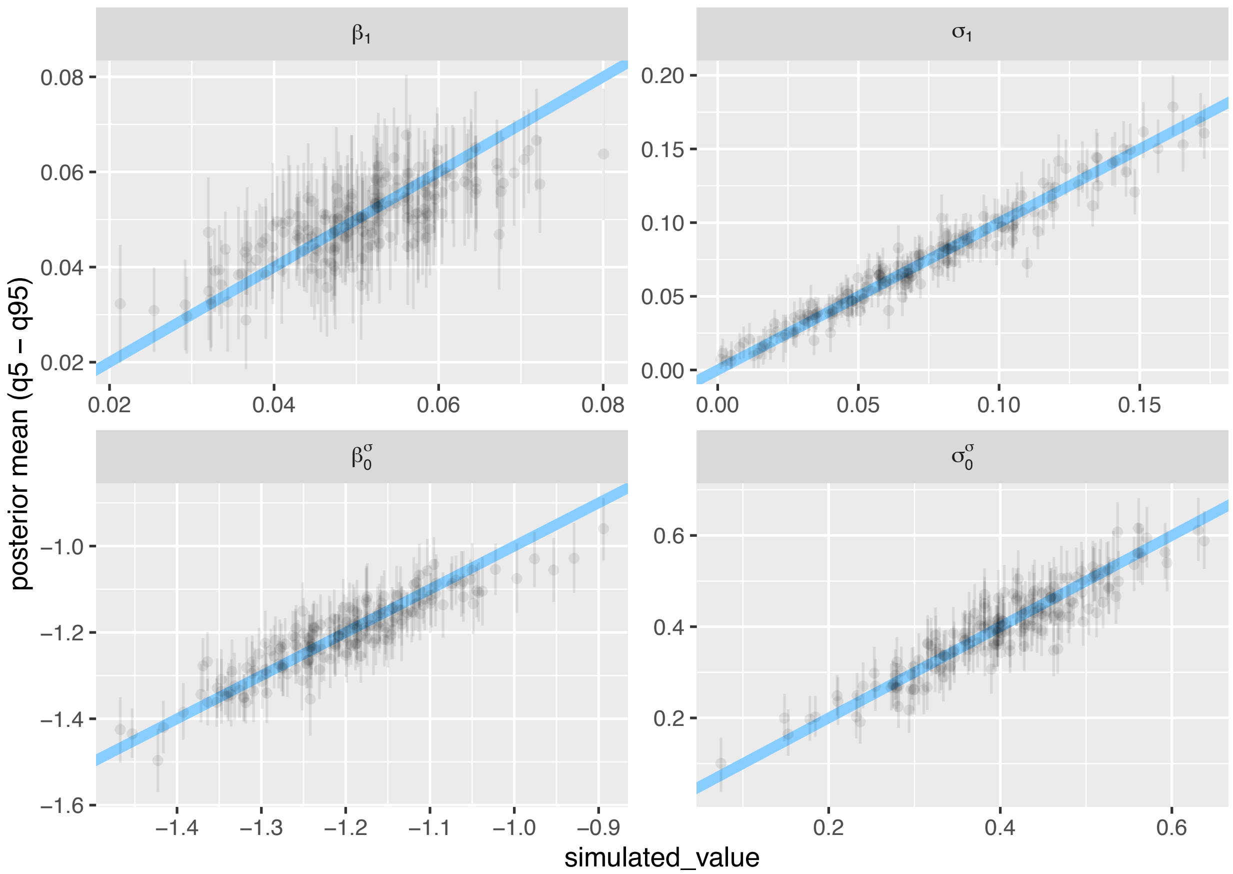

Figure S16: Model 2.4 was able to recover known parameter values with reasonable accuracy. Results are shown for n = 181 simulated data sets; "simulated_value" (x-axis) is the known value of the parameter for a given simulation. Each point shows a parameter estimate, whiskers show 95% credible interval; diagonal line is the 1:1 line.

Model 2.5

The final model extended model 2.3 to allow for correlations between all of the varying study-level parameters (i.e., for the location and the scale):

$$S_{ij} \sim lognormal(\mu_{ij}, \sigma_i^2)$$

$$\mu_{ij} = \beta_0 + \beta_{0i} + (\beta_1 + \beta_{1i})X_{ij},$$

$$log(\sigma_i) = \beta_0^\sigma + \beta_{0i}^\sigma + (\beta_1^\sigma + \beta_{1i}^\sigma)X_i,$$

$$[\beta_{0i}, \beta_{1i}, \beta_{0i}^\sigma, \beta_{1i}^\sigma]' \sim MVN(0, \boldsymbol{SRS}),$$

$$\boldsymbol{S} = \begin{bmatrix} \sigma_{0i} & 0 & 0 & 0 \\ 0 & \sigma_{1i} & 0 & 0 \\ 0 & 0 & \sigma_{0i}^{\sigma} & 0 \\ 0 & 0 & 0 & \sigma_{1i}^{\sigma} \end{bmatrix},$$

$$\boldsymbol{R} = \begin{bmatrix} 1 & \rho_{\sigma_{0i}\sigma_{1i}} & \rho_{\sigma_{0i}\sigma_{0i}^{\sigma}} & \rho_{\sigma_{0i}\sigma_{1i}^{\sigma}} \\ \rho_{\sigma_{0i}\sigma_{1i}} & 1 & \rho_{\sigma_{1i}\sigma_{0i}^{\sigma}} & \rho_{\sigma_{1i}\sigma_{1i}^{\sigma}} \\ \rho_{\sigma_{0i}\sigma_{0i}^{\sigma}} & \rho_{\sigma_{1i}\sigma_{0i}^{\sigma}} & 1 & \rho_{\sigma_{0i}^{\sigma}\sigma_{1i}^{\sigma}} \\ \rho_{\sigma_{0i}\sigma_{1i}^{\sigma}} & \rho_{\sigma_{1i}\sigma_{1i}^{\sigma}} & \rho_{\sigma_{0i}^{\sigma}\sigma_{1i}^{\sigma}} & 1 \end{bmatrix}.$$

I fit the model with weakly regularizing priors:

$$\beta_0 \sim N(2.5, 1),$$

$$\beta_1, \beta_0^{\sigma}, \beta_1^{\sigma} \sim N(0, 1),$$

$$[\sigma_{0i}, \sigma_{1i}, \sigma_{0i}^{\sigma}, \sigma_{1i}^{\sigma}] \sim N(0, 1),$$

$$\mathbf{R} \sim LKJ(1). \qquad \text{(Model 2.5)}$$

This model had good convergence (all Rhat < 1.01), and showed a reasonable fit to the empirical data (Appendix S2: Fig. S17).

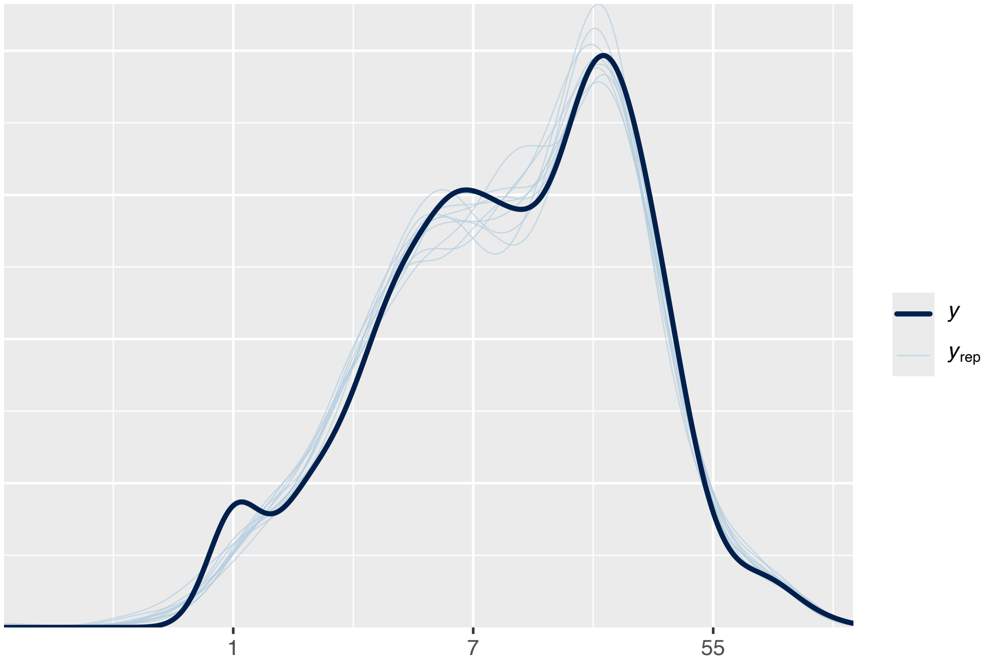

Figure S17: Model 2.5 made predictions that resembled the observed data closely.

The parameter estimates from the fit of model 2.5 to empirical data (β_0: 2.47 [95% credible interval: 2.3 – 2.66]; β_1: 0.05 [95% credible interval: 0.03 – 0.06]; σ_{0i}: 1.01 [95% credible interval: 0.89 – 1.15]; σ_{1i}: 0.06 [95% credible interval: 0.04 – 0.07]; β_0^σ: -1.28 [95% credible interval: -1.39 – -1.18]; β_1^σ: -0.06 [95% credible interval: -0.09 – -0.04]; σ_{0i}^σ: 0.46 [95% credible interval: 0.39 – 0.56], and σ_{1i}^σ: 0.07 [95% credible interval: 0.03 – 0.11]) were used to inform the following priors:

$$\beta_0 \sim N(2.5, 0.1),$$

$$\beta_1 \sim N(0.05, 0.01),$$

$$\sigma_{0i} \sim N(1, 0.1),$$

$$\sigma_{1i} \sim N(0.05, 0.05),$$

$$\beta_0^\sigma \sim N(-1.2, 0.1),$$

$$\sigma_{0i}^\sigma \sim N(0.4, 0.1).$$

Simulation-based calibration for model 2.5 showed that the rank statistics were approximately uniformly distributed (Appendix S2: Fig. S18), and that the coverage of parameters was reasonable (Appendix S2: Fig. S19); known parameters were approximately recovered (Appendix S2: Fig. S20).

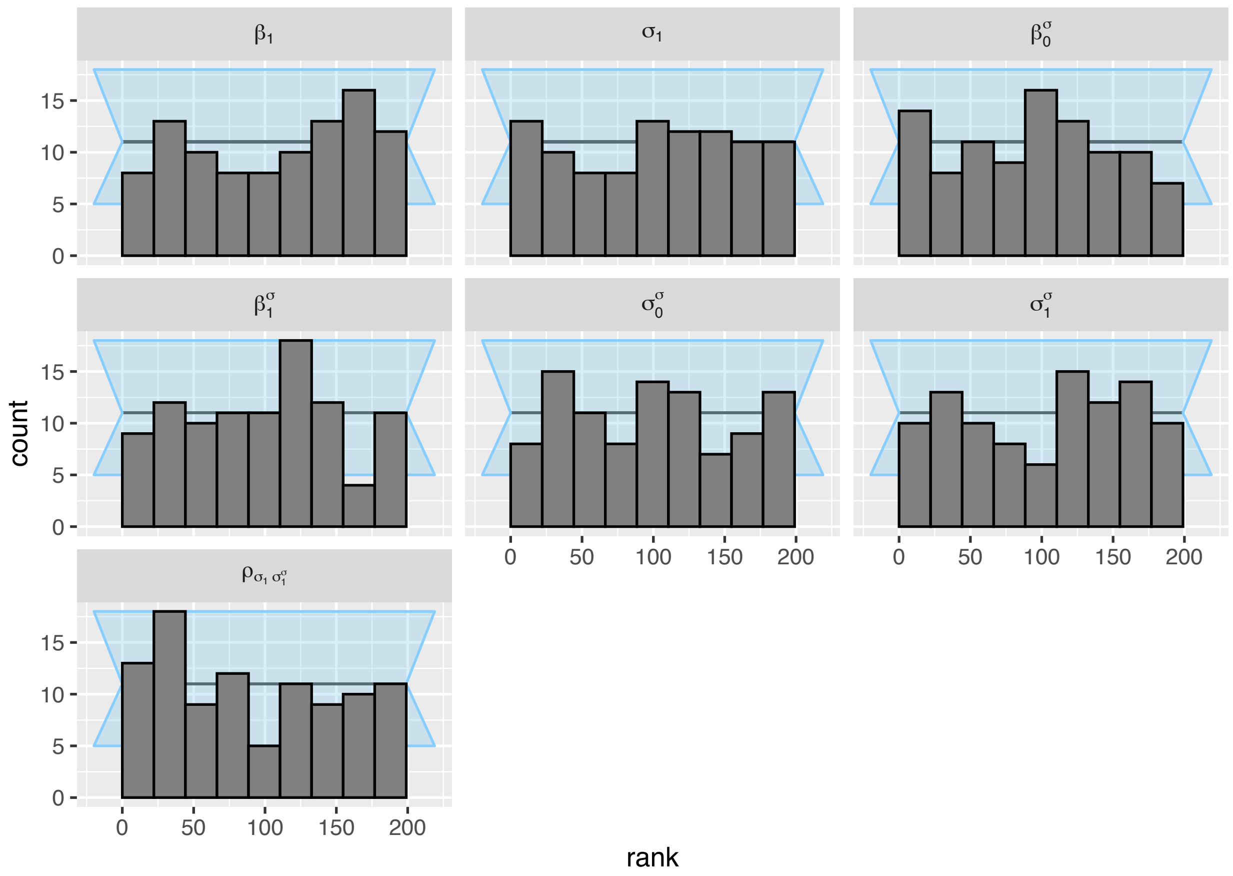

Figure S18: The posterior ranks of the prior draws were approximately normally distributed for the parameters of interest in model 2.5. Results are shown for n = 98 simulated data sets.

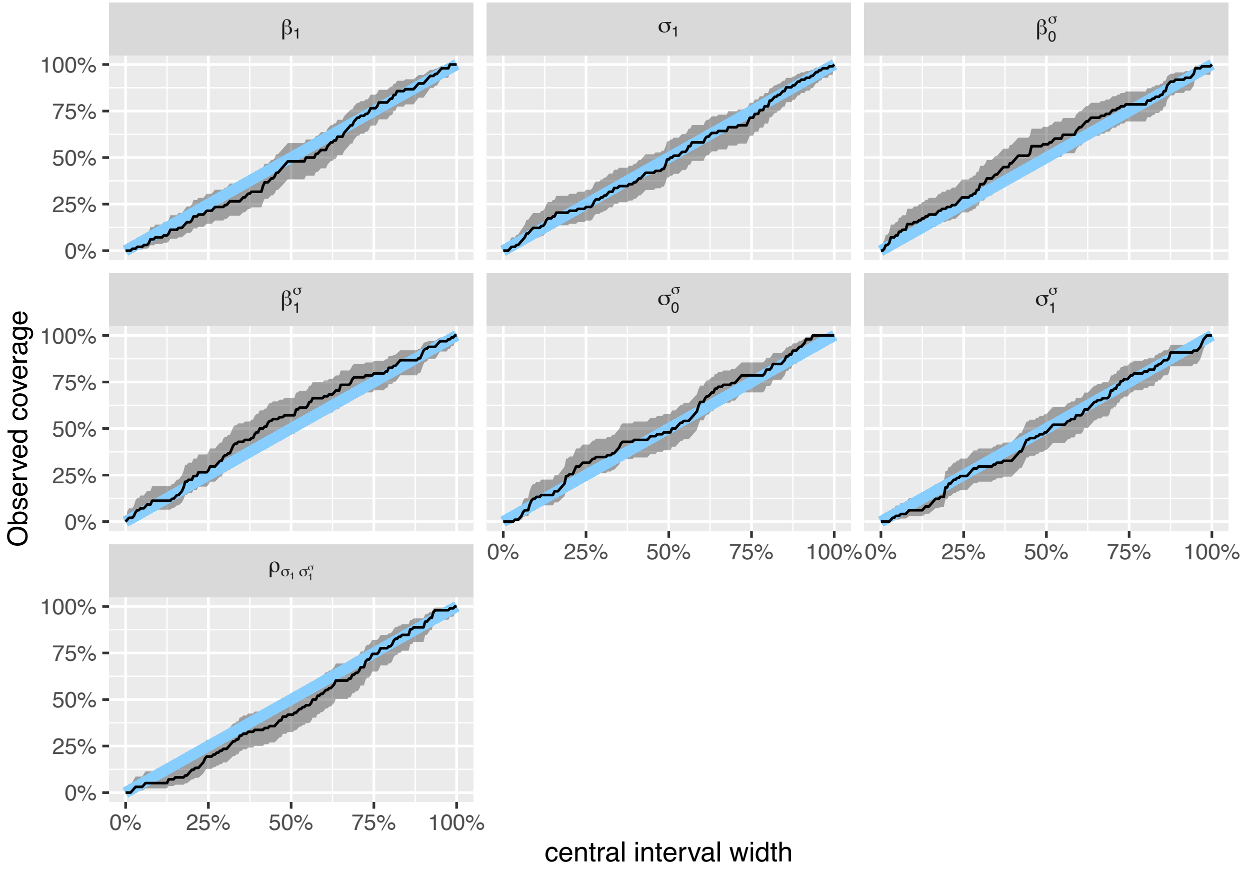

Figure S19: Model 2.5 had good coverage for all parameters of interest. Results are shown for n = 98 simulated data sets. Blue line is 1:1 line, and shading shows 95% uncertainty interval for the coverage.

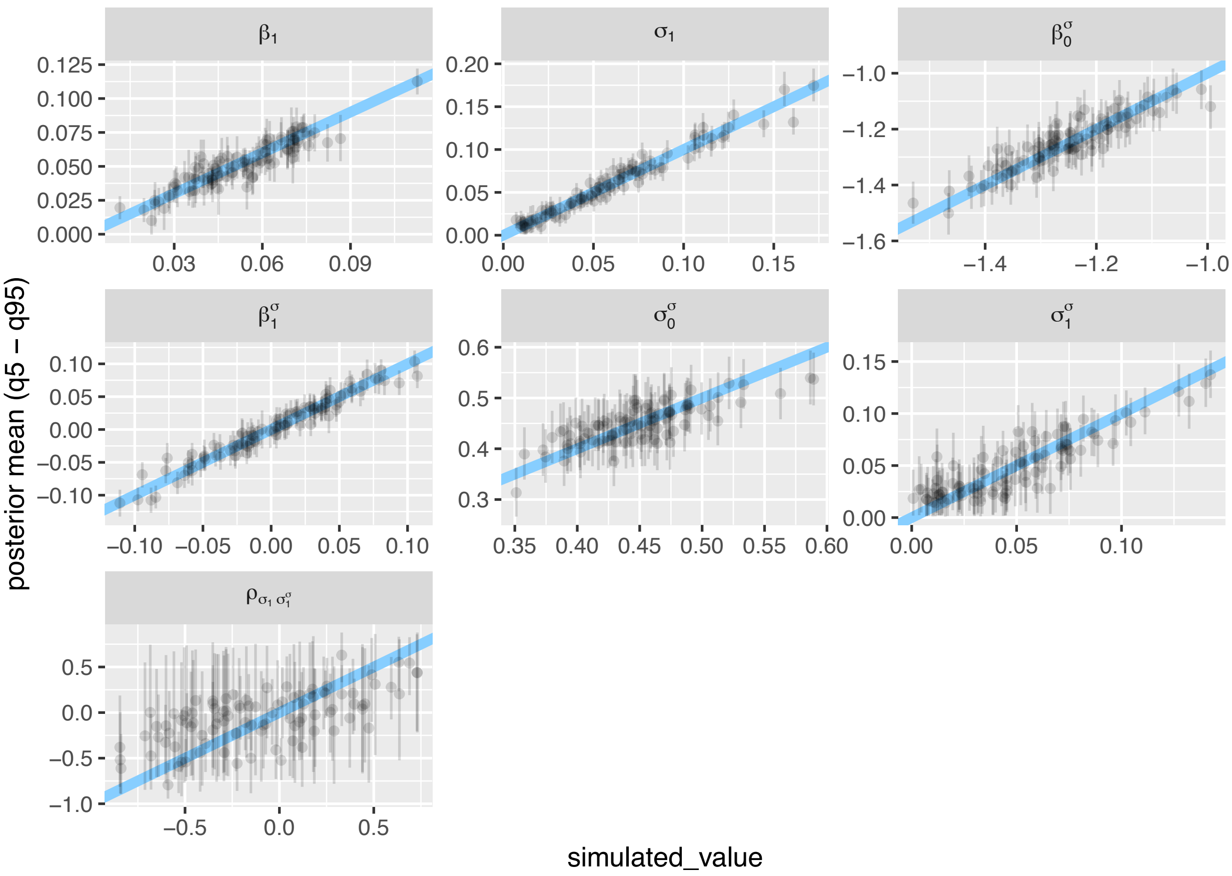

Figure S20: Model 2.5 was able to recover known parameter values with reasonable accuracy, with the exception of the correlation ($\rho_{\sigma_1\sigma_1^\sigma}$), which was estimated with high uncertainty (low precision). Results are shown for n = 98 simulated data sets; "simulated_value" (x-axis) is the known value of the parameter for a given simulation. Each point shows a parameter estimate, whiskers show 95% credible interval; diagonal line is the 1:1 line.

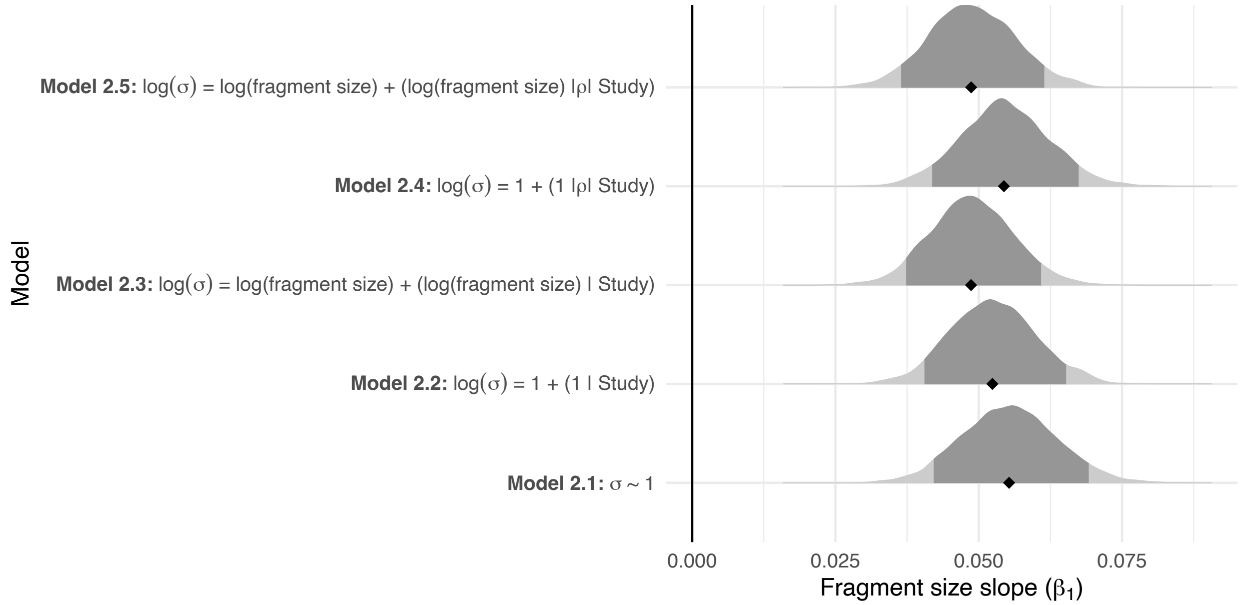

Figure S21: The different models for heteroscedasticity did not qualitatively change the support for the ecosystem decay hypothesis (i.e., posterior distributions of the slope parameter for the location [β_1] were greater than zero).

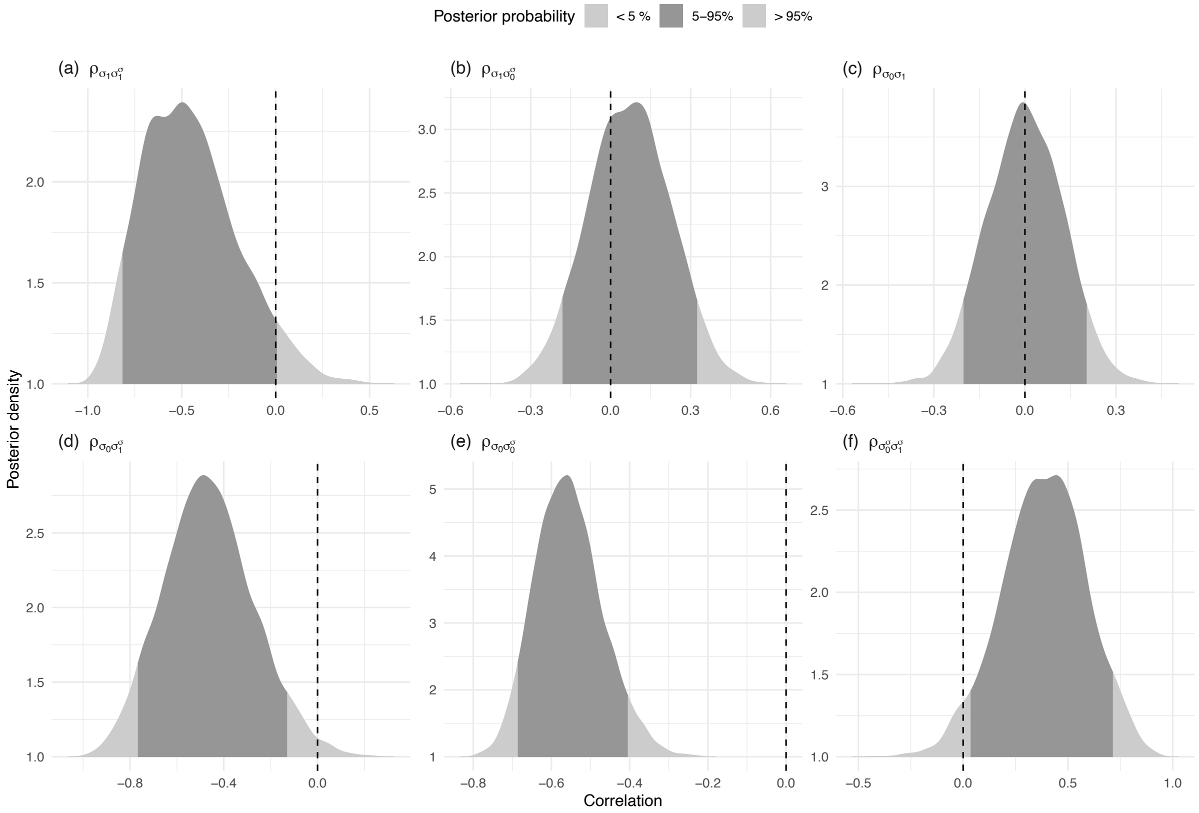

Figure S22: Posterior distributions (density plots for 1000 posterior draws) showing correlations among the varying study-level parameters for model 2.5.

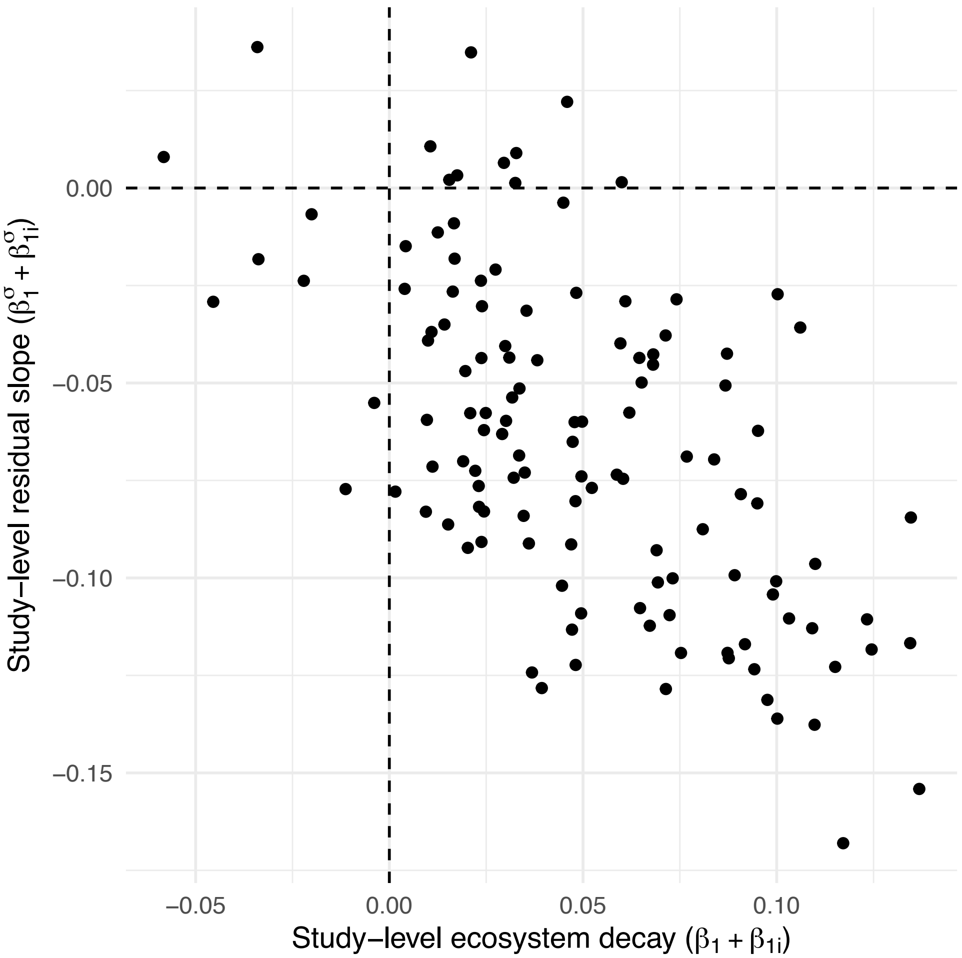

Figure S23: The decline of residual variation (scale) with increasing patch size was greatest for studies with the strongest ecosystem decay. Each point shows the estimate of a study-level slope, with slopes for the location on the x-axis, and the scale on the y-axis.

References

Chase, J. M., S. A. Blowes, T. M. Knight, K. Gerstner, and F. May. 2020. Ecosystem decay exacerbates biodiversity loss with habitat loss. Nature 584:238–243.

Modrák, M., A. H. Moon, S. Kim, P. Bürkner, N. Huurre, K. Faltejsková, A. Gelman, and A. Vehtari. 2023. Simulation-based calibration checking for Bayesian computation: The choice of test quantities shapes sensitivity. Bayesian Analysis 1:1–28.

Talts, S., M. Betancourt, D. Simpson, A. Vehtari, and A. Gelman. 2020, October 21. Validating Bayesian Inference Algorithms with Simulation-Based Calibration. arXiv.
